# Targeting the splicing factor CWC22 induces mitotic slippage through repression of BubR1 expression and CDK1 activity in cancer cells

**DOI:** 10.1016/j.jbc.2026.111148

**Published:** 2026-01-12

**Authors:** Ryuzaburo Yuki, Youhei Saito, Yuji Nakayama

**Affiliations:** Laboratory of Biochemistry and Molecular Biology, Kyoto Pharmaceutical University, Kyoto, Japan

**Keywords:** CWC22, RNA splicing, mitosis, cell cycle, checkpoint control, BubR1, cyclin-dependent kinase, mitotic slippage

## Abstract

Splicing factors play a fundamental role in gene expression. Several splicing factors are highly expressed in cancers and promote cell proliferation. Although targeting splicing factors prolongs the duration of G2/M phase, the involvement of splicing factors in the regulation of mitotic checkpoint signaling remains unclear. In this study, we found that knockdown of the splicing factor CWC22 increased not only the population of G2 phase and mitotic cells but also that of tetraploid cells. Notably, CWC22 knockdown induced mitotic slippage, which exhibited premature mitotic exit without spindle assembly checkpoint (SAC) satisfaction following prolonged prometaphase duration. CWC22 knockdown led to cyclin B1 degradation and accumulation of inactive cyclin-dependent kinase 1 with inhibitory phosphorylation at Tyr15 in mitosis. Simultaneous cyclin B1 overexpression and Wee1 blockade mitigated the shortened mitotic duration caused by CWC22 knockdown. RNA-Seq analysis indicated that CWC22 knockdown downregulated SAC-regulatory genes, including BubR1. The shortened mitotic duration caused by CWC22 knockdown was also mitigated by both overexpression of BubR1 and Wee1 blockade. Public datasets showed that CWC22 was highly expressed in pancreatic or cervical cancers, and higher expression negatively correlated with patient prognosis. Targeting CWC22 induced cancer cell death following mitotic slippage and a prolonged G2 phase because of DNA damage accumulation. These results suggest that highly expressed CWC22 contributes to the progression of G2/M phase and prevents mitotic slippage–caused whole-genome doubling by maintaining the SAC function and cyclin-dependent kinase 1 activity in cancer cells. These findings reveal a novel splicing factor function in mitotic checkpoint signaling, which enables uncontrolled cell proliferation in CWC22-overexpressing cancer cells.

Uncontrolled proliferation is a hallmark of cancer cells, and molecular pathways are rewired to sustain such proliferation (1, 2). Regarding mitotic processes, various mitotic regulators are highly expressed in cancer cells (3), and many cancer cells exhibit aneuploidy or whole-genome doubling (WGD), thereby contributing to error-prone mitosis through chromosomal instability (CIN) (4, 5). To prevent mitotic failures that lead to CIN, the spindle assembly checkpoint (SAC) monitors the proper mitotic progression (6). Until the kinetochore–microtubule attachment is completely established, SAC delays mitotic progression (7). SAC works through the formation of the mitotic checkpoint complex (MCC), which consists of BubR1, Mad2, Bub3, and Cdc20. MCC binds to the ubiquitin ligase anaphase-promoting complex/cyclosome (APC/C) and inactivates it, thereby inhibiting the degradation of target proteins, including cyclin B1, and preventing premature mitotic exit (8, 9). However, prolonged mitotic arrest or checkpoint dysfunction causes gradual APC/C activation and subsequent cyclin-dependent kinase 1 (CDK1) inactivation because of cyclin B1 degradation, leading to mitotic slippage, a phenomenon characterized by premature exit from mitosis without SAC satisfaction (10). On the other hand, CIN is thought to be a double-edged sword in cancer survival, and excessive CIN induces cancer cell death; therefore, CIN-promoting intervention may be useful for anticancer therapy (11, 12). We aimed to identify novel mitotic regulators in cancer cells by a small-scale siRNA screen and found the splicing regulator CWC22 as a candidate for a mitotic regulator.

RNA splicing is a fundamental process of mRNA maturation in eukaryotes (13). Splicing factors interact with the target pre-mRNAs step by step and remove intron sequences *via* catalytic reactions (14). Since the flow of transcription–splicing is needed to be smoothly progressed for proper gene expression, transcription and splicing are intrinsically coupled, and defects in splicing factors cause transcription abnormality (15, 16). Several splicing factors are highly expressed in cancer cells and support protein expression necessary for rapid proliferation; therefore, targeting splicing factors suppresses cancer cell proliferation, and the development of such interventions is considered promising for anticancer therapy (17, 18). Blockade of the splicing machinery is known to reduce mRNA levels of several mitotic genes (19) and induce mitotic catastrophe because of defects in sister chromatid cohesion (20, 21, 22). CWC22, one of the splicing factors, is known to regulate the cell cycle, and its targeting increases the population of 4C DNA cells through an unknown mechanism (23). CWC22 associates with the spliceosome before catalytic steps and promotes the first catalytic step *via* Prp2 helicase (24). Moreover, since CWC22 supports nonsense-mediated decay (NMD) *via* direct interaction with the exon junction complex (EJC) component eIF4AIII, CWC22 is thought to act as a hub protein between RNA splicing and NMD (25, 26, 27). Although CWC22 exerts an important role in RNA splicing (28), the precise mechanism of CWC22-mediated regulation of mitosis remains poorly understood.

In this study, we demonstrate that CWC22 knockdown induces defects in chromosome alignment and subsequent mitotic slippage (10). RNA-Seq and downstream analyses reveal that CWC22 knockdown causes mitotic slippage *via* both downregulation of BubR1 and an increase in the level of inhibitory phosphorylation of CDK1 at Tyr15. Furthermore, targeting CWC22 induces tetraploidization through mitotic slippage and also prolongs the duration of the G2 phase, eventually leading to cancer cell death. These results highlight a novel SAC-regulatory role of the splicing factor in cancer cells.

## Results

### The role of CWC22 in mitosis

**Figure 1:**
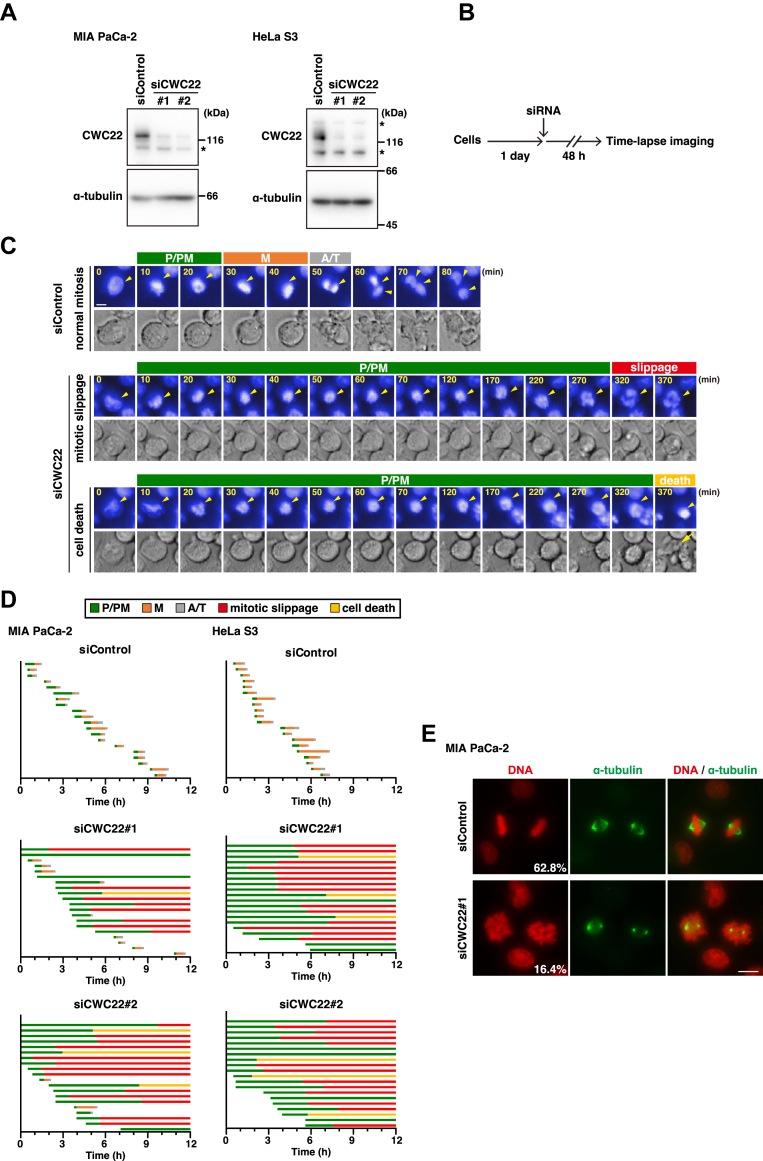
**CWC22 knockdown induces defects in chromosome alignment and mitotic slippage**. MIA PaCa-2 cells or HeLa S3 cells were transfected with control siRNA (siControl) or CWC22-targeting siRNAs (siCWC22#1 and #2). *A*, at 48 h after transfection, Western blot analysis was performed with the indicated antibodies. *Asterisks* show nonspecific bands. *B–D*, at 48 h after transfection, the cells were monitored for 12 h by time-lapse imaging with 0.1 μM Hoechst 33342. *B*, a schematic depiction of the time course of the experiment is shown. *C*, images show the typical phenotypes of siRNA-treated mitotic cells: cells that exhibit normal mitosis, cells that exhibit mitotic exit without SAC satisfaction after prolonged-prophase/prometaphase (P/PM) duration (mitotic slippage), and cells that exhibit chromosome condensation with membrane blebbing after prolonged-P/PM duration (cell death). *Yellow arrowheads* show the corresponding cells, and the *yellow arrow* shows the dead cells exhibiting a membrane bleb. The scale bar represents 10 μm. *D*, graphs show the individual cells (*y*-axis) and the duration of each subphase and the onset of mitotic slippage or cell death (*x*-axis): P/PM (chromosome condensation and congression, *green*), metaphase (M: chromosome alignment, *orange*), anaphase/telophase (A/T: from anaphase onset to cleavage furrow ingression, *gray*), mitotic slippage (*red*), and cell death (*yellow*). In total, 20 mitotic cells were examined. *E*, at 47 h after siRNA transfection, the cells were treated with 40 μM MG132 for 1 h, fixed, and stained for α-tubulin (*green*) and DNA (*red*). The scale bar represents 10 μm. The percentage of mitotic cells having aligned chromosomes is shown. SAC, spindle assembly checkpoint.

To investigate the role of CWC22 in mitosis, we knocked down CWC22 expression using two different siRNAs in human pancreatic cancer MIA PaCa-2 and cervical cancer HeLa S3 cells (Fig. 1*A*). We performed time-lapse imaging analysis and plotted each cell fate: the duration of each mitotic subphase and the onset of cell death and mitotic slippage, in which condensed chromosomes following mitotic entry decondensed again without cytokinesis (Fig. 1, *B*–*D*). In control cells, mitosis progressed properly and was completed within about 1 h: chromatin condensation, chromosome alignment, and chromosome segregation (Fig. 1, *C* and *D*, siControl). In contrast, more than half of CWC22-knockdown MIA PaCa-2 cells exhibited prolonged duration from chromosome condensation to alignment (Fig. 1, *C* and *D*, P/PM, *green*). Notably, 40% and 65% of cells treated with siCWC22#1 and siCWC22#2, respectively, underwent mitotic slippage after prolonged prometaphase duration (Fig. 1*D*, mitotic slippage, *red*). Mitotic cell death with membrane blebs was also observed in ∼15% of CWC22-knockdown MIA PaCa-2 cells (Fig. 1, *C* and *D*, cell death, *yellow*). Furthermore, mitotic slippage after prolonged prometaphase duration was also observed in 70% and 65% HeLa S3 cells treated with siCWC22#1 and siCWC22#2, respectively (Fig. 1*D*, HeLa S3). The proteasome inhibitor MG-132 prevents anaphase onset and stops the mitotic progression at metaphase by blocking the cyclin B1 degradation, as shown in our previous studies (29, 30). The percentage of chromosome-aligned metaphase cells was also decreased in CWC22-knockdown mitotic cells after short-term treatment with MG-132 (Fig. 1*E*), supporting the result of defects in chromosome alignment caused by CWC22 knockdown. These results suggest that CWC22 knockdown induces mitotic slippage after defects in chromosome alignment.

### CWC22 knockdown causes mitotic slippage through APC/C and CDK1 inactivation

**Figure 2:**
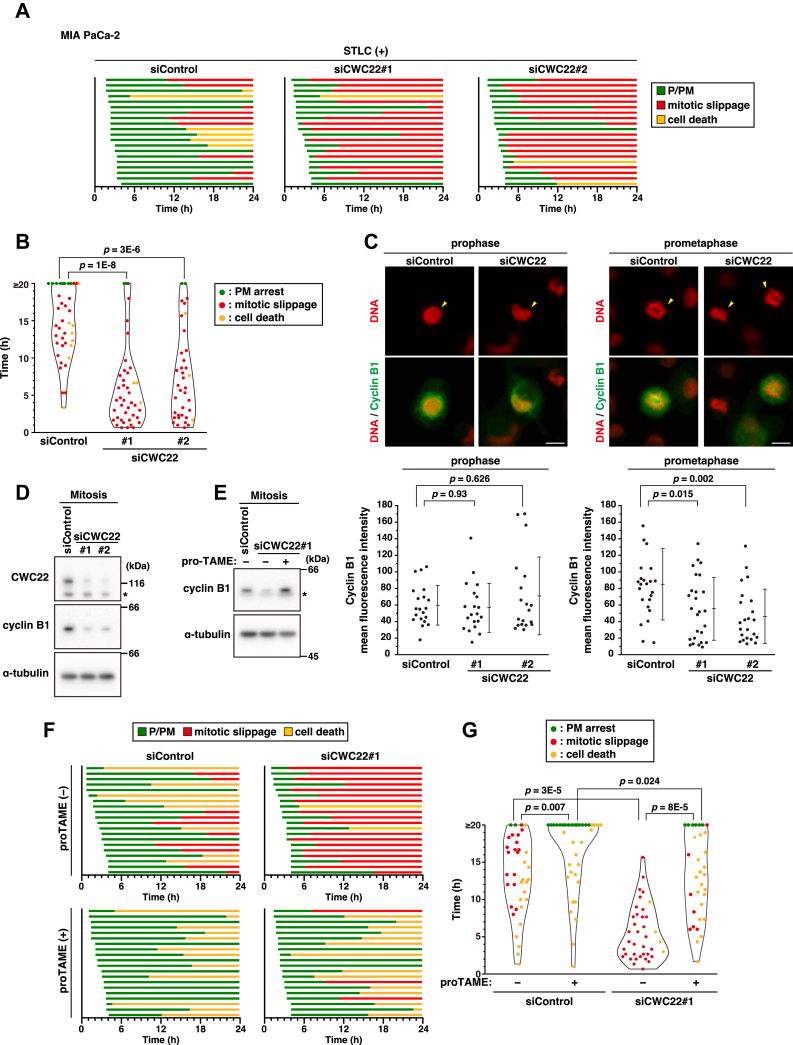
**CWC22 knockdown causes mitotic slippage through APC/C**. MIA PaCa-2 cells were transfected with control siRNA (siControl) or CWC22-targeting siRNAs (siCWC22#1 and #2). *A* and *B*, at 39 h after transfection, the cells were treated with 5 μM STLC for 1 h and monitored for 24 h by time-lapse imaging with 0.1 μM Hoechst 33342. *A*, the duration of each mitotic phase is shown as indicated in Figure 1*D*. In total, 20 mitotic cells were examined. *B*, the duration of mitosis until mitotic slippage (*red*) or cell death (*yellow*) was measured among the cells that entered mitosis within 4 h of starting time-lapse analysis is plotted as the mean ± SD from two experiments (n ≥ 37). Prometaphase-arrested cells where no events occurred over 20 h (*green*) are included in ≥20 h. *C*, at 40 h after siRNA transfection, the cells were fixed and stained for cyclin B1 (*green*) and DNA (*red*). Representative images are shown, and *yellow arrowheads* show the representative cells. The scale bars represent 10 μm. The mean fluorescence intensity of cyclin B1 within whole cells in prophase or prometaphase per cell was measured and plotted as the mean ± SD from a representative experiment (n = 20). *D*, at 36 h after transfection, cells were treated with 5 μM STLC for 12 h. The mitotic cells were collected *via* mitotic shake-off, and Western blot analysis was performed with the indicated antibodies. An *asterisk* shows a nonspecific band. *E*, at 36 h after transfection, cells were treated with 5 μM STLC with or without 5 μM proTAME for 12 h. The mitotic cells were collected *via* mitotic shake-off, and Western blot analysis was performed with the indicated antibodies. An *asterisk* shows a nonspecific band. *F* and *G*, at 39 h after transfection, the cells were treated with 5 μM STLC in the presence or absence of 5 μM proTAME for 1 h and monitored for 24 h by time-lapse imaging with 0.1 μM Hoechst 33342. *F*, the duration of each mitotic phase is shown as indicated in *A*. In total, 20 mitotic cells were examined. *G*, the duration of mitosis is shown as indicated in *B* (n ≥ 33). *P* Values were determined using the Steel test in *B*, the Games–Howell test in *C*, and the Steel–Dwass test in *G*. APC/C, anaphase-promoting complex/cyclosome; STLC, S-trityl-l-cysteine.

One of the causes of mitotic slippage is SAC defect–induced degradation of cyclin B1 and subsequent CDK1 inactivation even under the condition of SAC-mediated arrest at mitosis (10, 31). To scrutinize the effect of CWC22 knockdown on SAC, we treated cells with S-trityl-l-cysteine (STLC), which inhibits Eg5 motor protein and subsequently activates SAC. Indeed, STLC treatment exhibited prolonged mitosis at prometaphase and eventually led to mitotic slippage (50%) or cell death (30%), as previously indicated (32) (Fig. 2*A*, siControl). Upon CWC22 knockdown, mitotic slippage appeared to occur early after mitotic entry, and the percentage of mitotic slippage within the time-lapse observation was increased to 85% (Fig. 2*A*, siCWC22, mitotic slippage). We measured the mitotic duration among the STLC-arrested cells that entered mitosis within 4 h of starting time-lapse analysis and plotted the duration and cell fates: no events within the time-lapse observation (PM arrest, *green*), mitotic slippage (*red*), and cell death (*yellow*) (Fig. 2*B*). Although we observed a prolonged mitotic duration and some cells arrested in prometaphase for over 20 h in control cells, CWC22 knockdown clearly reduced the mitotic duration (Fig. 2*B*, siCWC22). CWC22 knockdown also shortened the mitotic duration in STLC-arrested cells after release from thymidine synchronization (Fig. S2). Furthermore, CWC22 knockdown did not affect the fluorescence intensity of cyclin B1 in prophase cells (Fig. 2*C*, prophase), which exhibited nuclear cyclin B1 accumulation unlike the nuclear exclusion of cyclin B1 in G2 phase (Fig. S1); however, the fluorescence intensity was decreased in CWC22 knockdown–prometaphase cells, and several prometaphase cells exhibited exceedingly low cyclin B1 expression (Fig. 2*C*, prometaphase). This result was confirmed by Western blot analysis in STLC-synchronized mitotic cells collected by shake-off (Fig. 2*D*). Considering that CWC22 does not affect the protein level of cyclin B1 at the onset of mitosis, these results suggest that CWC22 knockdown accelerates cyclin B1 degradation without affecting its transcription level. Prometaphase cells showing the low cyclin B1 expression might be immediately before mitotic slippage (Fig. 2*C*, prometaphase, siCWC22). Moreover, we treated cells with the APC/C inhibitor proTAME, and the treatment mitigated CWC22 knockdown–caused decrease in cyclin B1 expression (Fig. 2*E*). Consistent with this result, the percentage of mitotic slippage upon CWC22 knockdown was decreased from 90% to 15% by proTAME treatment (Fig. 2*F*, mitotic slippage), and the treatment prolonged mitotic duration in STLC-treated CWC22-knockdown cells (Fig. 2*G*, siCWC22). These results suggest that APC/C-mediated degradation of cyclin B1 is involved in CWC22 knockdown–caused mitotic slippage.

**Figure 3:**
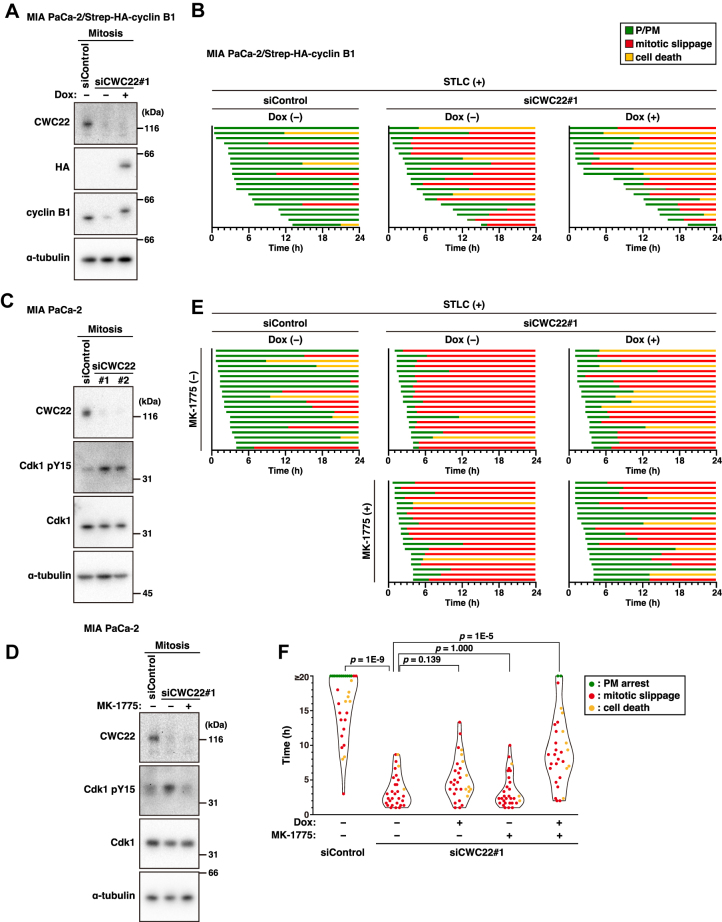
**Cyclin B1 degradation and inhibitory phosphorylation of CDK1 cooperatively contribute to CWC22 knockdown–induced mitotic slippage**. *A* and *B*, MIA PaCa-2/Strep-HA-cyclin B1 cells were transfected with control siRNA (siControl) or CWC22-targeting siRNAs (siCWC22#1) in the presence or the absence of 2 μg/ml doxycycline (Dox). *A*, 36 h after transfection, the cells were treated with 5 μM STLC for 12 h. The mitotic cells were collected *via* mitotic shake-off, and Western blot analysis was performed with the indicated antibodies. *B*, at 39 h after transfection, the cells were treated with 5 μM STLC for 1 h and monitored for 24 h by time-lapse imaging with 0.1 μM Hoechst 33342. The duration of each mitotic phase is shown as indicated in Figure 1*D*. In total, 20 mitotic cells were examined. *C*, MIA PaCa-2 cells were transfected with siControl, siCWC22#1, or siCWC22#2. About 36 h after transfection, the cells were treated with 5 μM STLC for 12 h. The mitotic cells were collected *via* mitotic shake-off, and Western blot analysis was performed with the indicated antibodies. *D*, MIA PaCa-2 cells were transfected with siControl or siCWC22#1. About 39 h after transfection, the cells were treated with 5 μM STLC in the presence or absence of 20 nM MK-1775 for 12 h. The mitotic cells were collected *via* mitotic shake-off, and Western blot analysis was performed with the indicated antibodies. *E* and *F*, MIA PaCa-2/Strep-HA-cyclin B1 cells were transfected with siControl or siCWC22#1 in the presence or absence of 2 μg/ml Dox. At 39 h after transfection, the cells were treated with 5 μM STLC for 1 h in the presence or absence of 20 nM MK-1775 and monitored for 24 h by time-lapse imaging with 0.1 μM Hoechst 33342. *E*, the duration of each mitotic phase is shown as indicated in *B*. *F*, the duration of mitosis until mitotic slippage (*red*) or cell death (*yellow*) is shown as indicated in Figure 2*B* (n = 30). *P* Values were determined using the Steel–Dwass test in *F*. CDK1, cyclin-dependent kinase 1; HA, hemagglutinin; STLC, S-trityl-l-cysteine.

Next, we examined the effect of cyclin B1 overexpression on slippage induction by CWC22 knockdown. Doxycycline (Dox)-mediated expression of cyclin B1 was observed in CWC22 knockdown–mitotic cells and was comparable to control cells (Fig. 3*A*). However, cyclin B1 overexpression hardly affected slippage induction in CWC22-knockdown cells (Fig. 3*B*): the average duration from mitotic entry to slippage was 304 min in Dox-untreated (n = 16) and 284 min in Dox-treated (n = 11) CWC22-knockdown cells. Previous reports have shown that prolonged arrest at mitosis leads to gradual repression of CDK1 activity by reactivation of Wee1 and subsequent accumulation of inhibitory phosphorylation at Tyr15 of CDK1, thereby causing mitotic slippage (33). CWC22 knockdown increased the level of CDK1 pY15 in mitotic cells (Fig. 3*C*), and treatment with the Wee1 inhibitor MK-1775 mitigated it (Fig. 3*D*). To examine the involvement of inhibitory phosphorylation of CDK1 in CWC22 knockdown–induced mitotic slippage, we treated cells with MK-1775 in addition to cyclin B1 overexpression. Mitotic slippage seemed to occur early after mitotic entry, even in cyclin B1 overexpression or MK-1775 treatment; however, cyclin B1 overexpression with MK-1775 treatment delayed the onset of mitotic slippage in CWC22-knockdown cells (Fig. 3*E*). Based on the measurements of mitotic duration, cyclin B1 overexpression plus MK-1775 treatment strongly prolonged the duration in STLC-treated CWC22-knockdown cells; however, the duration was only slightly prolonged by cyclin B1 overexpression alone and not by MK-1775 treatment alone (Fig. 3*F*). These results suggest that not only the degradation of cyclin B1 but also the inhibitory phosphorylation of CDK1 contribute to the CDK1 inactivation in CWC22 knockdown cells, thereby causing mitotic slippage.

### Splicing function of CWC22 in mitotic progression

**Figure 4:**
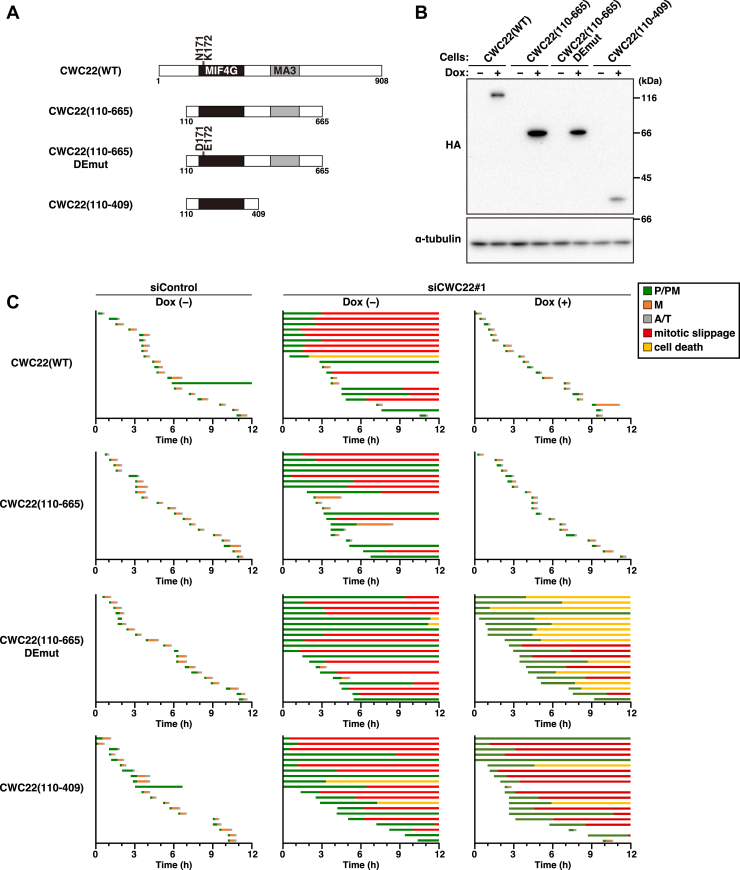
**The MA3 domain of CWC22 is important for mitotic regulation**. *A*, schematic depiction of the CWC22 domain structure and CWC22 mutants is shown. *B*, MIA PaCa-2/HA-CWC22(WT), CWC22(110–665), CWC22(110–665) DEmut, or CWC22(110–409) cells were treated with 1, 1, 1, or 2 μg/ml Dox for 48 h, respectively. Whole cell lysates were analyzed by Western blotting using the indicated antibodies. *C*, MIA PaCa-2 cells expressing inducible CWC22 wildtype or mutants were transfected with control siRNA (siControl) or CWC22-targeting siRNAs (siCWC22#1). At 48 h after transfection, the cells were monitored for 12 h by time-lapse imaging with 0.1 μM Hoechst 33342. The duration of each mitotic phase is shown as indicated in Figure 1*D*. In total, 20 mitotic cells were examined. Dox, doxycycline; HA, hemagglutinin.

CWC22 interacts with the spliceosome and the EJC and plays roles in general RNA splicing and NMD, respectively. However, a previous report has shown that NMD defects are not observed upon CWC22 knockdown, likely because of impaired pre-mRNA splicing (26). Therefore, to examine the role of the splicing function of CWC22 in mitosis, we constructed a spliceosome-binding–deficient mutant of CWC22 by deleting the MA3 domain [CWC22(110–409)] (Fig. 4*A*), as previously reported (27). The expression of CWC22(WT) induced by Dox treatment clearly mitigated the mitotic defects caused by CWC22 knockdown: the percentage of cells exhibiting prolonged prometaphase, mitotic slippage, or cell death was decreased from 75% to 0% upon CWC22(WT) re-expression (Fig. 4, *B* and *C*). Furthermore, the mitotic defects were similarly mitigated by the expression of the N-terminal and C-terminal deletion mutant of CWC22 [CWC22(110–665)], which possesses only the core MIF4G and MA3 domains, as previously reported (27). Notably, expression of the MA3 domain deletion mutant [CWC22(110–409)] did not mitigate the mitotic defects, which were observed in 100% of Dox-untreated and 85% of Dox-treated CWC22-knockdown cells, indicating the importance of the MA3 domain in CWC22’s mitotic regulation.

To examine whether the interaction between CWC22 and the EJC is dispensable for mitotic regulation, we constructed the splicing-competent but EJC-binding–deficient mutant of CWC22 [CWC22(110–665) DEmut], as previously described (26). In these mutant-expressing cells, defects in chromosome alignment and mitotic slippage were observed, similar to CWC22-knockdown cells (Fig. 4, *B* and *C*). However, mitotic cell death was strongly induced by the mutant expression: the percentage was increased from 10% of Dox-untreated to 60% of Dox-treated CWC22-knockdown cells [Fig. 4*C*, CWC22(110–665) DEmut, cell death]. It has been reported that the re-expression of the EJC-binding–deficient mutant of CWC22 upregulates NMD target genes, including SC35 and GAS5, because of the impaired NMD-mediated mRNA degradation (26). Overexpression of GAS5 is known to promote apoptosis and inhibit tumor growth (34, 35, 36), suggesting the possibility that the NMD abnormalities might induce mitotic defects and mitotic catastrophe. Collectively, these results suggest that CWC22’s splicing regulation contributes to mitotic progression in cancer cells.

### SAC gene maintenance by CWC22

**Figure 5:**
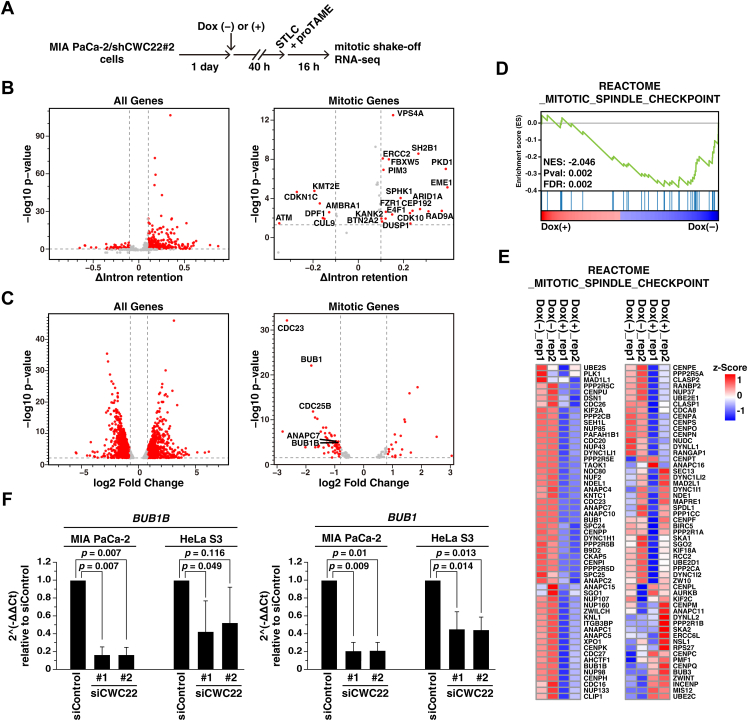
**CWC22 knockdown downregulates the mRNA levels of SAC-regulatory genes, including BubR1 and Bub1**. *A*–*E*, MIA PaCa-2/shCWC22#2 cells were treated with 4 μg/ml Dox for 40 h and then treated with 5 μM STLC plus 5 μM proTAME for 16 h. Mitotic cells were collected by mitotic shake-off and analyzed by RNA-Seq. *A*, a schematic depiction of the synchronization method is shown. *B* and *C*, volcano plot showing differential degree of intron retention (*B*) or differential gene expression (*C*) in Dox-treated cells compared with Dox-untreated cells. Representative upregulated and downregulated genes are labeled in each panel. *D*, GSEA plots for the gene set “REACTOME_MITOTIC_SPINDLE_CHECKPOINT” between Dox-treated and untreated cells are shown. *E*, heatmap of relative expression values (z-score of normalized read counts) from REACTOME_MITOTIC_SPINDLE_CHECKPOINT dataset (n = 2). *F*, MIA PaCa-2 cells were transfected with control siRNA (siControl) or CWC22-targeting siRNAs (siCWC22#1 and #2). At 44 h after transfection, the cells were treated with 5 μM STLC plus 5 μM proTAME for 16 h. The mitotic cells were collected *via* mitotic shake-off and analyzed by real-time PCR for *BUB1B* and *BUB1* mRNA. The fold changes in mRNA levels are expressed relative to siControl. *P* Values were determined using the Audic and Claverie test in *B*, the Wald's test in *C*, and the Games–Howell test in *F*. Dox, doxycycline; FDR, false discovery rate q value; GSEA, gene set enrichment analysis; NES, normalized enrichment score; SAC, spindle assembly checkpoint; STLC, S-trityl-l-cysteine.

To identify the target genes involved in CWC22 knockdown–mediated mitotic slippage, we performed RNA-Seq analysis in mitotic cells. We established cells expressing inducible shRNA targeting CWC22 (Fig. S3*A*), treated cells with STLC together with Dox plus proTAME, and efficiently collected mitotic cells even upon CWC22 knockdown (Fig. 5*A*). First, we analyzed splicing defects by CWC22 knockdown. We calculated the degree of intron retention (IR) in mRNAs using the IRFinder program (37), and found that CWC22 knockdown strongly increased IR in various mRNAs (Fig. 5*B*, *left*). We narrowed down the genes involved in mitotic regulation referenced by the Gene Ontology term “mitotic cell cycle” (38, 39); however, we hardly found genes that regulate SAC or mitotic slippage, among the intron-retained genes (Fig. 5*B*, *right*). Previous reports have shown that splicing machinery is functionally coupled to transcription, and thus splicing defects can cause transcription abnormalities (15, 16). Therefore, we analyzed differentially expressed genes after CWC22 knockdown and found altered levels of various mRNAs (Fig. 5*C*, *left*). Among the mitotic genes, the SAC-regulatory genes *BUB1B* and *BUB1* were downregulated by CWC22 knockdown (Fig. 5*C*, *right*). Furthermore, gene set enrichment analysis (GSEA) showed negative enrichment of the SAC gene set in CWC22-knockdown cells (Fig. 5*D*), and heatmap analysis showed that levels of various SAC genes, including *BUB1B* and *BUB1*, were decreased (Fig. 5*E*). These results suggest the possibility that CWC22 knockdown disrupts coupling between transcription and splicing machineries because of global splicing defects, thereby reducing the expression of SAC-regulatory genes. BubR1, encoded by the *BUB1B* gene, and Bub1 play central roles in SAC. Under SAC-activating conditions, Bub1 acts as a platform for the formation of the MCC, which consists of BubR1, Bub3, Mad2, and Cdc20, and the MCC suppresses the activity of APC/C (40). We confirmed that CWC22 knockdown repressed mRNA levels of *BUB1B* and *BUB1* in both MIA PaCa-2 and HeLa S3 cells (Fig. 5*F*). These results suggest that CWC22 contributes to maintaining the mRNA levels of SAC genes, including *BUB1B* and *BUB1*.

### The involvement of BubR1 downregulation in CWC22 knockdown–induced mitotic slippage

**Figure 6:**
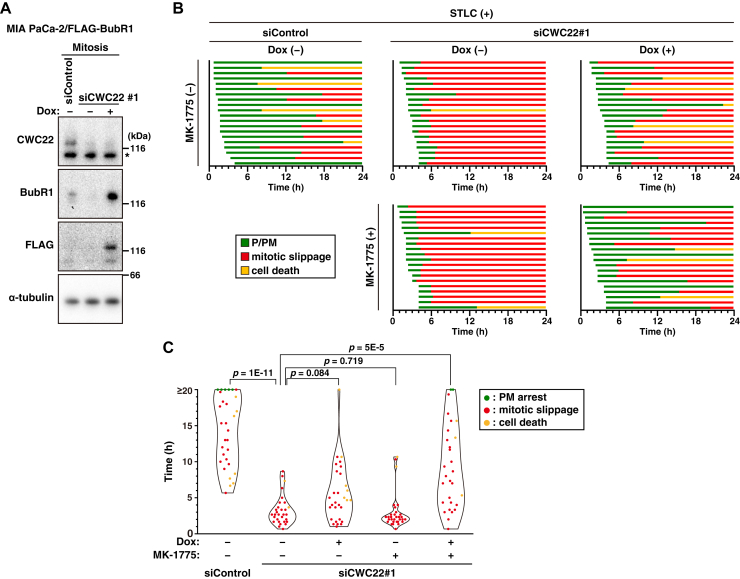
**CWC22 knockdown causes mitotic slippage through both downregulation of BubR1 and inhibitory phosphorylation of CDK1**. MIA PaCa-2/FLAG-BubR1 cells were transfected with control siRNA (siControl) or CWC22-targeting siRNAs (siCWC22#1) in the presence or absence of 0.5 μg/ml Dox. An *asterisk* shows a nonspecific band. *A*, 36 h after transfection, the cells were treated with 5 μM STLC for 12 h. The mitotic cells were collected *via* mitotic shake-off, and Western blot analysis was performed with the indicated antibodies. *B* and *C*, at 39 h after transfection, the cells were treated with 5 μM STLC for 1 h in the presence or absence of 20 nM MK-1775 and monitored for 24 h by time-lapse imaging with 0.1 μM Hoechst 33342. *B*, the duration of each mitotic phase is shown as indicated in Figure 1*D*. In total, 20 mitotic cells were examined. *C*, the duration of mitosis until mitotic slippage (*red*) or cell death (*yellow*) is shown as indicated in Figure 2*B* (n ≥ 29). *P* Values were determined using the Steel–Dwass test in *C*. CDK1, cyclin-dependent kinase 1; Dox, doxycycline; STLC, S-trityl-l-cysteine.

To examine whether downregulation of BubR1 or Bub1 is involved in mitotic slippage upon CWC22 knockdown, we established Dox-inducible cell lines of FLAG-tagged BubR1 and hemagglutinin (HA)-tagged Bub1. In MIA PaCa-2/FLAG-BubR1 cells, CWC22 knockdown reduced the BubR1 protein level, and Dox treatment induced BubR1 overexpression (Fig. 6*A*). Time-lapse imaging analysis showed that the onset of mitotic slippage appeared to be delayed upon Dox treatment in STLC-treated CWC22-knockdown cells (Fig. 6*B*). Indeed, Dox treatment alone tended to prolong the mitotic duration (Fig. 6*C*). We further treated cells with MK-1775 to activate CDK1, and the additional MK-1775 treatment significantly prolonged mitotic duration (Fig. 6, *B* and *C*). On the other hand, expression of HA-tagged Bub1 did not affect mitotic duration in STLC-treated CWC22-knockdown cells (Fig. S4). These results suggest that CWC22 knockdown causes mitotic slippage through downregulation of BubR1, besides accumulation of inhibitory phosphorylation of CDK1.

### The effect of CWC22 knockdown on cell cycle progression

**Figure 7:**
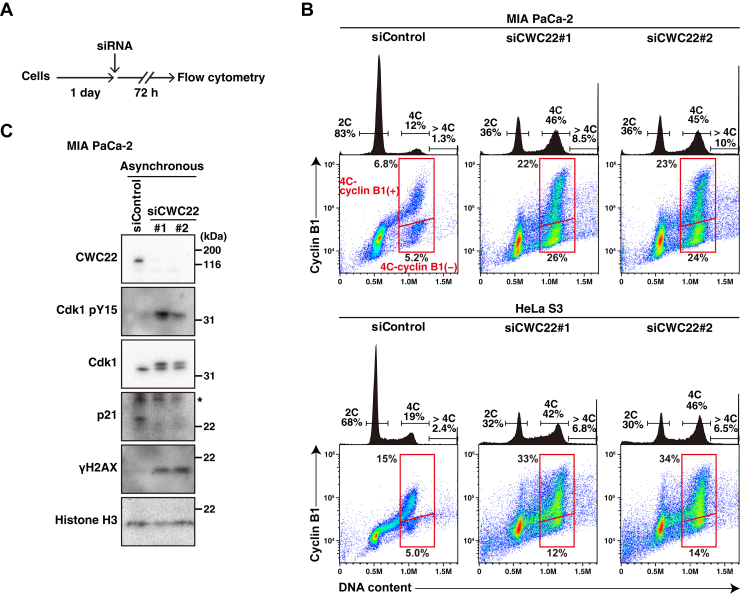
**CWC22 knockdown increases the population of G2/M cells and tetraploid cells**. MIA PaCa-2 cells or HeLa S3 cells were transfected with control siRNA (siControl) or CWC22-targeting siRNAs (siCWC22#1 and #2). *A* and *B*, at 72 h after transfection, the cells were fixed, doubly stained with anti-cyclin B1 and propidium iodide (PI), and subsequently analyzed by flow cytometry. *A*, a schematic depiction of the time course of the experiment is shown. *B*, the bivariate dot plots of cyclin B1 protein level (*y*-axis, log scale) and DNA content (*x*-axis, linear scale) are shown together with DNA histograms. The ratios of 2C, 4C, polyploid cells (>4C), and cells having 4C DNA content with or without cyclin B1 (*red line*) are indicated. Each plot represents 40,000 cells. *C*, at 60 h after transfection, Western blot analysis was performed with the indicated antibodies. An *asterisk* shows a nonspecific band.

We examined the effect of CWC22 knockdown on the entire cell cycle. Flow cytometry (FC) analysis showed that the percentage of 4C cells was strongly increased upon CWC22 knockdown (Fig. 7, *A* and *B*), consistent with a previous report (23). Interestingly, among 4C cells, CWC22 knockdown not only increased the percentage of 4C cells with higher cyclin B1 expression but also that of 4C cells with lower cyclin B1 expression, indicating that CWC22 knockdown increased both G2/M-phase cells and 4N-G1 cells. Moreover, the percentage of >4C cells that include proliferating 4N cells and polyploid cells was also increased upon CWC22 knockdown. The tetraploidy induction by CWC22 knockdown was consistent with the result of mitotic slippage (Fig. 1). Previous reports have shown that defects in splicing factors prolong the duration of G2 phase through accumulation of DNA damage (23). Consistent with this, CWC22 knockdown increased the γH2AX level, a marker of DNA damage (Fig. 7*C*). Moreover, the inhibitory phosphorylation of CDK1 at Tyr15, but not p21 expression, was increased by CWC22 knockdown in asynchronous cells.

**Figure 8:**
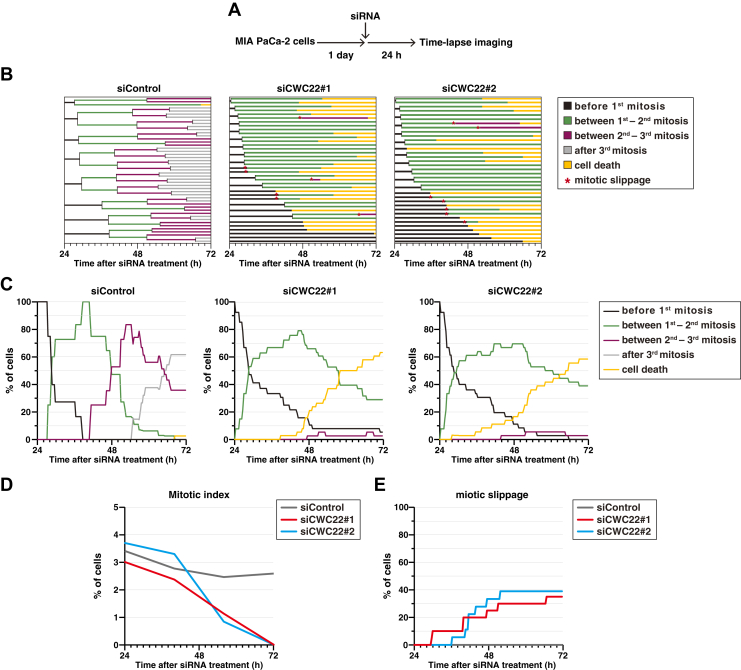
**CWC22 knockdown exhibits cell death after prolonged interphase and mitotic slippage**. MIA PaCa-2 cells were transfected with siControl, siCWC22#1, and siCWC22#2. At 24 h after transfection, the cells were monitored for 72 h by time-lapse imaging with 0.05 μM Hoechst 33342. *A*, a schematic depiction of the time course of the experiment is shown. *B*, the duration of each phase is presented: before first mitosis (*black*), between first–second mitosis (*green*), between second–third mitosis (*purple*), after third mitosis (*gray*), cell death (*yellow*), and mitotic slippage (*asterisk*). *C*, the percentages of each phase at the indicated times are plotted. *D*, the percentages of mitosis at 24, 48, and 72 h after siRNA treatment are plotted. *E*, the percentages of mitotic slippage among total mitosis within the time-lapse observation are cumulatively plotted.

Furthermore, we performed long-term time-lapse imaging analysis (Fig. 8*A*). Control cells exhibited proper cell cycle progression and division (Fig. 8, *B* and *C*, siControl). In the early stage of CWC22 knockdown, mitotic entry occurred similarly to control cells, and the mitotic index was comparable, although some cells showed prolonged interphase and mitotic slippage (Fig. 8, *B*–*E*, siCWC22). Meanwhile, in the late stage of CWC22 knockdown, the frequency of mitotic entry decreased, but the limited number of M-phase cells exhibited a high frequency of mitotic slippage. Moreover, most cells eventually underwent cell death. Collectively, these results suggest that CWC22 knockdown promotes tetraploidization through mitotic slippage and prolongs the duration of the G2 phase because of DNA damage accumulation, resulting in cancer cell death.

### Highly expressed CWC22 contributes to cancer cell proliferation

**Figure 9:**
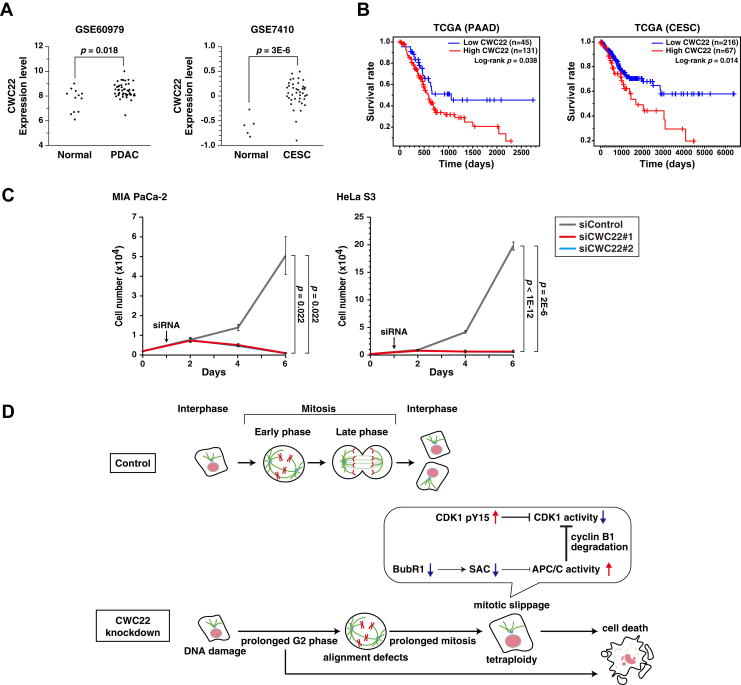
**Targeting the highly expressed CWC22 strongly decreases the viable number in cancers**. *A*, the relative mRNA expression of CWC22 in normal pancreatic tissue and pancreatic ductal adenocarcinoma (PDAC) tissue (*left*) and normal cervical tissue and cervical squamous cell carcinoma (CESC) tissue (*right*) in GSE60979 and GSE7410 datasets are shown, respectively. *B*, pancreatic adenocarcinoma (PAAD) patients (*left*) and CESC patients (*right*) in TCGA datasets were divided into two groups based on CWC22 expression level, and Kaplan–Meier curves for overall survival are shown. *C*, MIA PaCa-2 cells or HeLa S3 cells were transfected with control siRNA (siControl) or CWC22-targeting siRNAs (siCWC22#1 and #2). After siRNA transfection, the cells were further cultured for 5 days. Cell numbers were counted 2, 4, and 6 days after seeding, and viable cell numbers were plotted. *D*, model of the role of CWC22 in cell cycle progression in cancer cells. Control cells exhibit proper cell cycle progression. CWC22 knockdown prolongs G2 phase duration because of the DNA damage accumulation. CWC22-knockdown cells entering mitosis undergo defects in chromosome alignment and subsequent mitotic slippage because of the APC/C activation and CDK1 inactivation through both BubR1 downregulation and accumulation of inhibitory phosphorylation of CDK1. Eventually, cells undergoing prolonged interphase or mitotic slippage exhibit cell death. APC/C, anaphase-promoting complex/cyclosome; CDK1, cyclin-dependent kinase 1; TCGA, The Cancer Genome Atlas.

Our results indicate the significance of CWC22 in cell cycle progression, suggesting its contribution to uncontrolled cancer growth. We analyzed the expression levels of CWC22 in cancer tissues, and its expression was higher in pancreatic ductal adenocarcinoma and cervical squamous cell carcinoma tissues (Fig. 9*A*). Furthermore, The Cancer Genome Atlas analysis showed that higher expression of CWC22 negatively correlated with patient prognosis (Fig. 9*B*). Indeed, CWC22 knockdown strongly suppressed the proliferation of pancreatic cancer MIA PaCa-2 cells and cervical cancer HeLa S3 cells (Fig. 9*C*). These results suggest the possibility that higher expression of CWC22 in pancreatic and cervical cancers contributes to the aberrant cancer proliferation by sustaining cell cycle progression.

## Discussion

In this study, we demonstrated that knockdown of the splicing regulator CWC22 induces defects in chromosome alignment and subsequent mitotic slippage, thereby leading to WGD. CWC22 knockdown also prolongs G2-phase duration through DNA damage accumulation. Moreover, CWC22 knockdown decreases the mRNA levels of SAC-regulatory genes, including BubR1, and increases the inhibitory phosphorylation of CDK1 in mitosis, thereby causing mitotic slippage. CWC22 expression is elevated in pancreatic and cervical cancer cells, and targeting CWC22 strongly represses cancer cell proliferation following prolonged G2-phase duration and mitotic slippage (Fig. 9*D*).

The absence of microtubule–kinetochore attachment activates the SAC and subsequently promotes the assembly of the MCC, including BubR1, thereby inhibiting APC/C-mediated ubiquitination of cyclin B1 (41). In the case of prolonged mitosis, APC/C activity gradually increases because of the incomplete suppression by SAC, leading to progressive cyclin B1 degradation (42). Once cyclin B1 level is reduced below the threshold, the residual CDK1 activity cannot maintain the mitotic state, resulting in mitotic slippage (43, 44). Furthermore, prolonged mitosis reactivates Wee1 kinase and subsequently reduces CDK1 activity through inhibitory phosphorylation at Tyr15, thereby promoting mitotic slippage (33). From our RNA-Seq and quantitative PCR analyses, the mRNA levels of BubR1 among SAC-regulatory genes were strongly decreased (Fig. 5). Interestingly, Wee1 inhibition combined with overexpression of BubR1 significantly prolonged mitotic duration in CWC22-knockdown cells, although BubR1 overexpression alone slightly prolonged it (Fig. 6*C*). Furthermore, Wee1 inhibition combined with cyclin B1 overexpression also prolonged mitotic duration (Fig. 3*F*). These results suggest a model in which CWC22 knockdown causes weak suppression of SAC function through BubR1 downregulation and possibly leads to APC/C activation. In turn, cyclin B1 degradation together with inhibitory phosphorylation of CDK1 might cooperatively inactivate CDK1, resulting in mitotic slippage (Fig. 9*D*). Since RNA-Seq analysis showed that Cdc25B, a CDK1 phosphatase, was downregulated by CWC22 knockdown (Fig. 5*C*, *right panel*), dephosphorylation of CDK1 at Tyr15 might be repressed, resulting in suppression of CDK1 activity.

CWC22 knockdown reduces mRNA levels of SAC-regulatory genes, including *BUB1B* and *BUB1* (Fig. 5). Knockdown of spliceosome subunits SNW1 and PRPF8 induces defects in the splicing machinery and increases the *CDCA5* mRNA with an unremoved intron 1 region, leading to reduced protein expression of CDCA5 and disruption of chromatid cohesion (21). Similarly, CWC22 knockdown increased reads within the intron 1 region of *CDCA5* (Fig. S3*B*, CDCA5). In contrast, the intron reads were hardly observed within the *BUB1B* and *BUB1* genes in CWC22-knockdown cells (Fig. S3*B*, BUB1B, BUB1), suggesting that *BUB1B* and *BUB1* might not be direct splicing targets of CWC22. The reduced mRNA levels of SAC-regulatory genes might be caused by splicing defects in upstream genes that regulate the expression of SAC-regulatory genes. Alternatively, the EJC, which binds to CWC22, is reported to regulate the proper transcription by directly modulating RNA polymerase II pausing, independently of pre-mRNA splicing (45); therefore, CWC22 might also be a component of the transcription machinery and contribute to the temporal transcription of SAC-regulatory genes. To precisely reveal how CWC22 regulates the expression of SAC-regulatory genes, further studies will be required. For example, the use of acute protein depletion strategies beyond siRNA-mediated knockdown is important to distinguish direct effects from secondary transcriptional or splicing-dependent events. In addition, comprehensive analyses of downstream gene expression after CWC22 protein depletion and CWC22-associated protein complexes will be important to clarify how CWC22 integrates splicing- and transcription-related functions to regulate mitotic gene expression.

CWC22 knockdown induced defects in chromosome alignment and prolonged mitotic duration (Fig. 1). Previous reports have shown that knockdown of splicing factors reduces expression of sororin, a regulator of sister chromatid cohesion, *via* mis-splicing-mediated mRNA degradation and thereby induces defects in cohesion (20, 21). However, cohesion-regulatory genes were not among the intron-retained or downregulated mRNAs (Fig. 5, *B* and *C*). GSEA showed that centrosome-related genes were downregulated by CWC22 knockdown (Fig. S3*C*). Following mitotic entry, mature centrosomes promote spindle microtubule assembly and bipolar spindle formation, thereby supporting chromosome alignment (46); therefore, CWC22 knockdown might weaken centrosome biogenesis or maturation, resulting in chromosome misalignment.

CWC22 knockdown also prolonged interphase duration and led to the accumulation of G2/M-phase cells (Fig. 7*B*), indicating the prolongation of G2-phase duration. Loss of splicing factors is known to increase R-loop formation and impair the expression of DNA damage repair genes, leading to arrest at G2-phase *via* replication stress and DNA damage accumulation (23, 47). Consistent with this, Western blot analysis showed that the γH2AX level was increased by CWC22 knockdown (Fig. 7*C*). CWC22 knockdown increased the CDK1 pY15 level, possibly through activation of the DNA damage checkpoint machinery, including Wee1 kinase. Furthermore, GSEA indicated that CWC22 knockdown decreased the mRNA levels of DNA damage repair genes (Fig. S3*D*), similar to the other splicing factors (23, 47). These results suggest that CWC22 knockdown–induced defects in DNA damage repair pathways, along with R-loop formation, lead to the accumulation of DNA damage and subsequent suppression of CDK1 activity, thereby causing a delay in G2-phase progression.

We found that CWC22 is highly expressed in pancreatic and cervical cancers, and that CWC22 knockdown represses cancer cell proliferation following chromosome misalignment and subsequent mitotic slippage–mediated WGD, accompanied by downregulation of SAC-regulatory genes without IR (Fig. 9). Several splicing factors are highly expressed in certain cancers and contribute to their proliferation (48), and this is consistent with the notion that cancer cells require highly efficient transcription–splicing coupling for rapid proliferation. Moreover, although CIN caused by WGD or aneuploidy can lead to stochastic carcinogenesis, excessive CIN could paradoxically induce cancer cell death (49), consistent with the “just-right” model of CIN in cancers (50). Collectively, these results suggest the possibility that rewiring the higher expression of CWC22 supports transcription of target genes required for cancer cell division, thereby preventing excessive CIN and contributing to cancer progression.

In conclusion, we demonstrate that targeting CWC22 efficiently induces cancer cell death following disruption of cell cycle progression, and this might be partly because of the induction of excessive CIN. Targeting highly expressed splicing factors in cancers has been reported to strongly repress cancer progression, similar to the effects observed upon CWC22 depletion (51, 52), and various splicing inhibitors have been developed and are currently in clinical trials (17, 53); therefore, CWC22-targeting agents might also be a promising therapeutic strategy for pancreatic ductal adenocarcinoma or cervical squamous cell carcinoma. Since CWC22 knockdown leads to both DNA damage accumulation and mitotic abnormalities, DNA-damage or microtubule-targeting agents might potentiate the cytotoxic effect of CWC22 intervention. Furthermore, re-expression of the EJC-binding–deficient mutant caused stronger cell death than CWC22 knockdown alone (Fig. 4*C*); therefore, targeting NMD machinery might also be an efficient anticancer therapy. Further studies are required to reveal the clinical significance of targeting CWC22 in cancers.

## Experimental procedures

### Cells

MIA PaCa-2 (RIKEN BRC), HeLa S3 (Japanese Collection of Research Bioresources), and Lenti-X 293T (Clontech Laboratories) cells were cultured in Dulbecco’s modified Eagle’s medium containing 10%, 5%, and 5% fetal bovine serum with 20 mM Hepes–NaOH (pH 7.4) at 37 °C in 5% CO_2_, respectively.

### Plasmids

Human CWC22 complementary DNA (cDNA) was obtained from Lenti-X 293T cells by PCR, fused to the N-terminal HA tag, and inserted into the pENTR4-no-ccDB (686-1) vector (a gift from Eric Campeau and Paul Kaufman [Addgene plasmid #17424; http://n2t.net/addgene:17424; Research Resource Identifier [RRID]: Addgene_17424]) (54) with the start codon removed from the multicloning site [pENTR4-no-ccDB (upATG−)]. The siRNA-resistant CWC22 construct was created by PCR-mediated introduction of a silent mutation into the siCWC22#1 target site with (sense) 5′-TCTAGAAAATCCCCATCTCCTGGGAGG-3′ and (antisense) 5′-GCGCTTCCGATCCGTATCTCTTTCTCTTTCTC-3′ [pENTR4_HA-CWC22(WT)]. The N-terminal deletion mutant of CWC22 was generated [pENTR4_HA-CWC22(110–908)] by inverse PCR from the template pENTR4_HA-CWC22(WT) plasmid with (sense) 5′-CAGGATGAACCTGCTACAAAGAAAAAGAAAGATGAG-3′ and (antisense) 5′-GGCGTAGTCGGGCACGTC-3′. The deletion mutants of CWC22 were generated by inverse PCR from the template pENTR4_HA-CWC22(110–908) plasmid using primers as follows: CWC22(110–665), (sense) 5′-TAATCTAGACCCAGCTTTCTTGTACAAAG-3′ and (antisense) 5′-TTTATTTTGCTCAACATCTGGTTTCTGC-3′; CWC22(110–409), (sense) 5′-TAATCTAGACCCAGCTTTCTTGTACAAAG-3′ and (antisense) 5′-TCCCTCATCAAGAATTTCTTTCTTAATAGCTTTG-3′. The mutant CWC22 with the Asn171 to Asp substitution (N171D) and Lys172 to Glu substitution (K172E) was generated [CWC22(110–665) DEmut] by inverse PCR from the template pENTR4_HA-CWC22(110–665) plasmid with (sense) 5′-GTTAATATTTCCAACATAAGTATTATTATTCAAGAGC-3′ and (antisense) 5′-TTCATCGATAAGGCCATTAATTGACTTCTTC-3′. These plasmids were recombined with pLIX_402 (gifted by David Root, plasmid 41394; Addgene) (55) lentiviral plasmids using the Gateway left–right reaction according to the manufacturer’s instructions (Thermo Fisher Scientific). pDONR223-BUB1B (Addgene plasmid #23858; http://n2t.net/addgene:23858; RRID: Addgene_23858) and pDONR223-BUB1 (Addgene plasmid #23612; http://n2t.net/addgene:23612; RRID: Addgene_23612) were gifts from William Hahn and David Root (56). BUB1B and BUB1 cDNAs were fused to the N-terminal FLAG and HA tags and inserted into the pENTR4-no-ccDB (upATG−) vector, respectively. The BUB1B plasmid was recombined with pLIX_402 as indicated above. The BUB1 plasmid was recombined with PB-TA-ERN piggyBac transposon Destination vector (gifted by Knut Woltjen, Addgene plasmid #80474; http://n2t.net/addgene:80474; RRID: Addgene_80474) (57) using the Gateway left–right reaction according to the manufacturer’s instructions (PB-TA-ERN_HA-BUB1). Cyclin B1 cDNA was obtained from a human cyclin B1-MmGFP construct (provided by J. Pines) (58) by PCR, fused to the N-terminal strep-HA tag, and inserted into the pENTR4-no-ccDB (upATG−) vector. Then, this plasmid was recombined with PB-TA-ERP2 piggyBac transposon Destination vector (gifted by Knut Woltjen, Addgene plasmid #80477; http://n2t.net/addgene:80477; RRID: Addgene_80477) (57) using the Gateway LR reaction according to the manufacturer’s instruction (PB-TA-ERP2_strep-HA-cyclin B1).

### RNA interference

MIA PaCa-2 cells and HeLa S3 cells were transfected with 10 pmol/well of siRNA in a 24-well plate or 1.25 pmol/well of siRNA in a 96-well plate using the Lipofectamine 2000 reagent (Thermo Fisher Scientific). siCWC22#1 (5′-CGGAAAAGGTCTCGGAAAT-3′) and CWC22#2 (5′-CAACAGAGGACATACGAAA-3′) were synthesized by MilliporeSigma. The oligonucleotides for CWC22 shRNA (shCWC22#2 [sense] 5′-CCGGCAACAGAGGACATACGAAATTCAAGAGATTTCGTATGTCCTCTGTTGTTTTTGCTAGC-3′ and [antisense] 5′-AATTGCTAGCAAAAACAACAGAGGACATACGAAATCTCTTGAATTTCGTATGTCCTCTGTTG-3′) were annealed and inserted into the Tet-pLKO-puro vector (Tet-pLKO-puro/shCWC22#2) (a gift from Dmitri Wiederschain [Addgene plasmid #21915; http://n2t.net/addgene:21915; RRID: Addgene_21915]) (59).

### Establishment of inducible cell lines *via* lentiviral transduction

To establish the Dox-inducible cell lines of CWC22, BUB1B, and CWC22 shRNA, Lenti-X 293T cells were cotransfected with 0.8 μg of pCAG-HIVgp, 0.8 μg of pCMV-VSV-G-RSV-Rev, and 1.2 μg of pLIX_402 vector harboring either construct or Tet-pLKO-puro/shCWC22#2 using PEIMAX (Polysciences) in a 35-mm dish. The medium was changed the next day after transfection, and the virus-containing medium was harvested 48 h after medium change and passed through a 0.45-mm filter. MIA PaCa-2 cells were infected with 80 μg/ml polybrene (MilliporeSigma) for 2 consecutive days and selected in 2 μg/ml puromycin (StressMarq Biosciences). To establish the Dox-inducible cell lines of BUB1 or cyclin B1, MIA PaCa-2 cells were cotransfected with 420 ng of PB-TA-ERN_HA-BUB1 or PB-TA-ERP2_strep-HA-cyclin B1 and 80 ng of Super PiggyBac Transposase vector (V012800; NovoPro Bioscience) using Lipofectamine 2000 in a 24-well plate. At 2 days after transfection, the cells expressing inducible BUB1 and cyclin B1 were selected in 1 mg/ml G418 and 1 μg/ml puromycin, respectively.

### Chemicals

The Eg5 inhibitor STLC (MilliporeSigma) was used at 5 μM. The proteasome inhibitor MG132 (3175-v; Peptide Institute) was used at 40 μM. The APC/C inhibitor proTAME (HY-124955; MedChemExpress; SML3977; MilliporeSigma) was used at 5 μM. The Wee1 inhibitor MK-1775 (CS-0105, ChemScene) was used at 20 nM.

### Antibodies

The following primary antibodies were used for immunofluorescence (IF), immunoblotting (IB), and FC: rat monoclonal anti-α-tubulin (IF, 1:800 dilution; IB, 1:4000 dilution; MCA78G; Bio-Rad), rabbit polyclonal anti-CWC22 (IB, 1:1000 dilution; 26898-1-AP, Proteintech Group, Inc), rabbit polyclonal anti-cyclin B1 (IF, 1:250 dilution; IB, 1:2000 dilution; FC, 1:250 dilution; sc-752, Santa Cruz Biotechnology), mouse monoclonal anti-HA-tag (IB, 1:1000 dilution; M180-3, Medical and Biological Laboratories), mouse monoclonal anti-Cdc2 p34 (IB, 1:500 dilution; sc-54, Santa Cruz Biotechnology), rabbit monoclonal anti-phospho-Cdc2 (Tyr-15) (1:500 dilution; #4539, Cell Signaling Technology), rabbit polyclonal anti-BUB1B (IB, 1:2000 dilution; 11504-2-AP, Proteintech Group, Inc), mouse monoclonal anti-FLAG (IB, 1:1000 dilution; F1804, MilliporeSigma), mouse monoclonal anti-p21 (IB, 1:400 dilution; #2946, Cell Signaling Technology), rabbit polyclonal anti–phospho-Histone H2A.X (Ser139) (IB, 1:500 dilution; #2577, Cell Signaling Technology), goat polyclonal anti-Histone H3 (IB, 1:400 dilution; sc-8654, Santa Cruz Biotechnology) antibodies. We validated the antibodies for IF based on the appropriate fluorescence signal of the protein at the previously reported localization. We validated the antibodies for IB based on the correct band size of the protein or the decrease in the level of the protein band following knockdown. For IF, Alexa Fluor 488–labeled donkey anti-rabbit and anti-rat (1:800 dilution; Life Technologies) immunoglobulin G (IgG) antibodies were used. For IB, horseradish peroxidase–conjugated anti-mouse (1:8000 dilution; 715-035-151), anti-rabbit (1:8000 dilution; 711-035-152), anti-rat (1:8000 dilution; 712-035-153), and anti-goat (1:8000 dilution; 705-035-147) IgG antibodies were purchased from Jackson ImmunoResearch. For FC, Alexa Fluor 488–labeled donkey anti-rabbit IgG antibody was used.

### Cell-cycle synchronization

STLC, an inhibitor of the Eg5 motor protein, was used for cell cycle synchronization in mitosis. Mitotic cells were collected by mitotic shake-off for use in Western blotting. To examine the effect of CWC22 knockdown on mitotic slippage under synchronization conditions, cells were treated with 4 mM thymidine (MilliporeSigma) for 20 h. After a 7-h release from thymidine, cells were treated with STLC for 1 h and subjected to time-lapse imaging. To analyze the effects of knockdown of CWC22 on chromosome alignment, CWC22-knockdown cells were treated with 40 μM MG132 for 60 min to prevent the onset of anaphase, fixed, and subjected to IF staining.

### IF microscopy

IF staining was performed as previously described (60). Briefly, formaldehyde-fixed cells were permeabilized and blocked with PBS(−) containing 0.1% saponin and 3% bovine serum albumin (BSA) for 30 min, incubated with the primary antibody for 1 h, and subsequently with the secondary antibody for 1 h along with 1 μM Hoechst 33342 for DNA staining. The fluorescence images were obtained using an IX-83 fluorescence microscope (Olympus) equipped with a 40×/0.45 numerical aperture or a 60×/1.42 numerical aperture oil-immersion objective lens (Olympus). The optical system included a U-FUNA filter cube (360–370 nm excitation, 420–460 nm emission) and a U-FBNA cube (470–495 nm excitation, 510–550 nm emission) to observe the Hoechst 33342 and Alexa Fluor 488 fluorescence, respectively. The captured images were edited using ImageJ (National Institutes of Health), Photoshop CC, and Illustrator CC software (Adobe). The mean fluorescence intensity of cyclin B1 in Figure 2 was measured by enclosing the entire cell using the ImageJ software.

### Time-lapse imaging

Time-lapse imaging analysis was performed as previously described (61). Briefly, regarding the short-term analysis, MIA PaCa-2 or HeLa S3 cells were transfected with siRNAs and treated with inhibitors as described in the figure legends. The cells were then treated with 0.1 μM Hoechst 33342, and time-lapse imaging was performed using a high-content imaging system (Operetta; PerkinElmer Life Sciences) at 37 °C in 5% CO_2_. Regarding the long-term analysis, cells were transfected with siRNAs as described in the figure legends. One day after transfection, cells were treated with 0.05 μM Hoechst 33342, and time-lapse imaging was performed using a BZ-X810 fluorescence microscope (Keyence) at 37 °C in 5% CO_2_.

### Western blotting

Western blotting was performed as previously described (62). Briefly, the cells were lysed in SDS sample buffer containing protease inhibitors (10 μg/ml aprotinin [Fujifilm Wako Pure Chemicals], 4 μg/ml pepstatin A [Peptide Institute, Inc], 10 μg/ml leupeptin [Nacalai Tesque], 2.5 mM EGTA–KOH (MilliporeSigma), and 1 mM PMSF [Nacalai Tesque]). The whole cell lysates were subjected to SDS-PAGE and electrotransferred onto polyvinylidene difluoride membranes (Pall Corporation). Blocking to minimize nonspecific interactions was done with Blocking One (Nacalai Tesque), 5% skim milk, or 5% BSA in Tween 20-containing Tris-buffered saline (20 mM Tris–HCl [pH 7.5], 137 mM NaCl, and 0.1% Tween-20) at room temperature for 30 min. Later, the membranes were incubated with the antibodies, which were diluted with Tween 20-containing Tris-buffered saline containing 5% Blocking One, 5% skim milk, or 5% BSA. Clarity (Bio-Rad) or Chemi-Lumi One Ultra (catalog no. 11644; Nacalai Tesque) was used as the chemiluminescence substrate. A ChemiDoc XRSplus image analyzer (Bio-Rad) was used for the chemiluminescence detection and band intensity analysis.

### RNA-Seq

Mitotic cells were collected by mitotic shake-off. Total RNA was purified using NucleoSpin RNA Plus (740984; Takara Bio) and subjected to RNA-Seq by Rhelixa, Inc. In brief, mRNA was isolated using the NEBNext Poly(A) mRNA Magnetic Isolation Module (New England Biolabs), and a cDNA library for sequencing was prepared using NEBNext Ultra II Directional RNA Library Prep Kit (New England Biolabs). Finally, samples were subjected to sequencing at 150 bp x2 by Illumina NovaSeq X Plus. The raw data have been deposited on the Gene Expression Omnibus database (accession number: GSE310789). Reads were mapped with STAR (version 2.7.10a) to ENSEMBL genome assembly GRCh37.87, indexed with SAMtools, and features counted with featureCounts. Differential expression was analyzed on the gene level with DESeq2 (63) and visualized as a volcano plot. GSEA was conducted with GSEApy (version 0.9.8) (64) using the preranked list of genes derived from DESeq2 analysis. GSEApy’s prerank function was used with standard parameters, except min_size, max_size,m and permutation whose values were set to 10, 1,000, and 10,000, respectively. Gene expression values were calculated as count per million reads, and a heatmap was visualized using the calculated z-score. IR was detected and characterized using IRFinder (version 1.3.1) (37). Introns with IR ratio >10%, intron depth >4, and splicing exact >10 were defined as being retained, as previously described (65). Differential IR between samples was analyzed using the Audic and Claverie test, and an absolute difference in the IR ratio (ΔIR = IR_Dox(+)_ − IR_Dox(−)_) was calculated.

### Real-time PCR

Total RNAs were isolated using the Sepasol-RNA I Super G (Nacalai Tesque), and cDNAs were prepared from RNAs using the ReverTra Ace qPCR RT Master Mix (TOYOBO). We used the following primers for PCR: BUB1, (sense) 5′-CGGCGGCTTCTAGTTTGCGG-3′ and (anti-sense) 5′-TGTGGGCTTCAAGCATCTGAAGGA-3′; BUB1B (BubR1), (sense) 5′-AGGACCAGCAGACAGCTTGTG-3′ and (anti-sense) 5′-AAGCCAGAGGAGTGTGTGGC-3′; and 18S rRNA, (sense) 5′-TGCGCCGCTAGAGGTGAAATT-3′ and (anti-sense) 5′-TGGCAAATGCTTTCGCTCT-3′. Quantitative PCR was performed using the Thunderbird Next SYBR qPCR Mix (TOYOBO) and QuantStudio 1 (Thermo Fisher Scientific) with an initial denaturation (30 s at 95 °C), denaturation (40 cycles of 5 s at 95 °C), and annealing/extension (10 s at 64 °C). 18S rRNA was used as an endogenous control, and the relative gene expression was evaluated using the ΔΔCt method.

### Flow cytometry

To collect dead and detached cells, floating cells in culture supernatants were collected and mixed with cells detached by trypsinization. The cells were fixed with 70% ethanol at −30 °C for 1 h. After washing the cells with PBS(−) containing 3% calf serum plus 0.1% Triton X-100, the cells were incubated at room temperature with anti-cyclin B1 antibody and subsequently with Alexa Fluor 488-labeled donkey anti-rabbit IgG antibody in PBS(−) containing 3% calf serum plus 0.1% Triton X-100 for 1 h in the dark. Then, the cells were stained for DNA with 50 μg/ml propidium iodide plus 200 μg/ml RNase A at 37 °C for 30 min in the dark. Immediately, the stained cells were analyzed using a flow cytometer equipped with a 488 nm solid-state blue laser (BD Accuri C6 Plus; BD Biosciences). Debris was excluded based on the forward and side scatter profiles. FlowJo software (Tree Star) was used for data analysis and plotting.

### Proliferation assay

Cells seeded in a 96-well plate were transfected with siRNAs after 1 day of culture and subsequently cultured for 5 days. Cells were trypsinized on 2, 4, and 6 days of culture, and viable cell numbers were counted.

### Statistics

Statistical differences between the two datasets were analyzed using Student's *t* test or Welch's *t* test after analysis of variance by *F* test. The parametric statistical tests among more than two datasets were performed using one-way ANOVA with Tukey's *post hoc* test or Dunnett's *post hoc* test, or Welch's ANOVA with Games–Howell *post hoc* test, depending on their variance that was analyzed using Bartlett's test. The nonparametric statistical tests among more than two datasets were performed using Kruskal–Wallis test with Steel or Steel–Dwass *post hoc* test. Statistical analysis was performed using EZR software (version 1.55; Saitama Medical Center, Jichi Medical University) (66) and R software (version 4.1.2; R Foundation for Statistical Computing). Statistical differences of the degree of IR were analyzed using the IRFinder program with Audic and Claverie tests. Statistical differences of the differentially expressed genes were analyzed using the DESeq2 library with Wald's test. Overall survival was analyzed by the Kaplan–Meier method, and the log-rank test was used to compare survival distributions between groups. The data analysis was conducted using Python (version 3.12.11) within the Google Colab environment.

## Data availability

The data used to support the findings of the study are available from the corresponding author upon reasonable request.

## Supporting information

This article contains supporting information.

## Conflict of interest

The authors declare that they have no conflicts of interest with the contents of this article.
