# Supplementary material for: Targeting the splicing factor CWC22 induces mitotic slippage through repression of BubR1 expression and CDK1 activity in cancer cells
Source: J Biol Chem. 2026 Jan 12;302(3):111148. doi: 10.1016/j.jbc.2026.111148 (PMC12887411; doi:10.1016/j.jbc.2026.111148)
Supplement: Supplementary Material 1 [file mmc1.pdf]

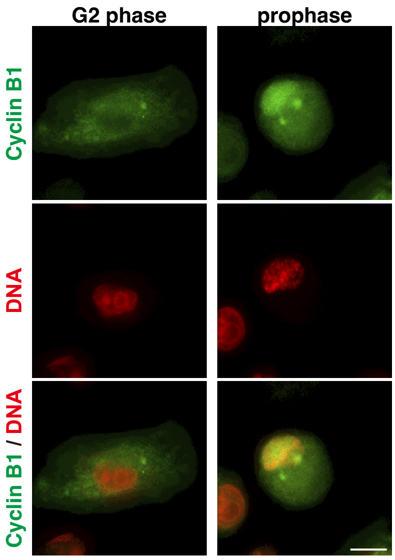

Figure S1

A

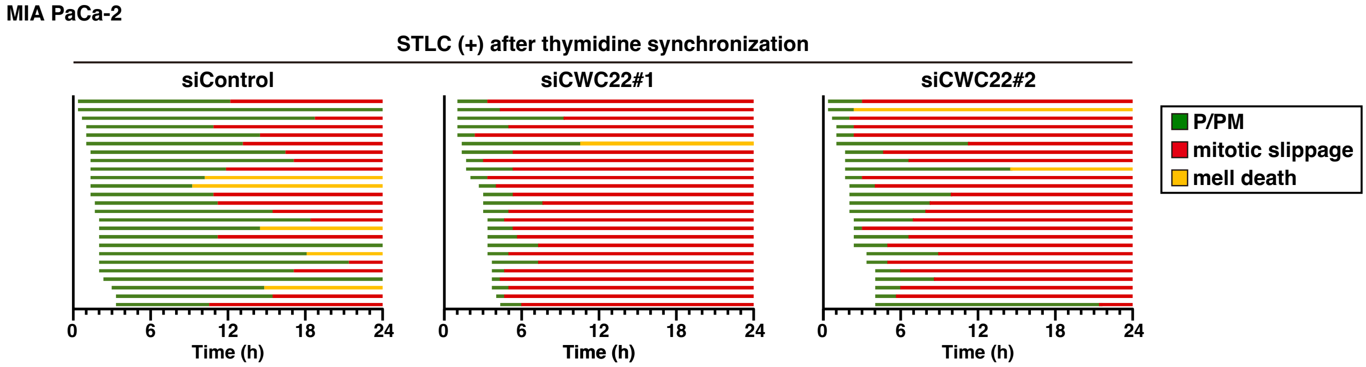
MIA PaCa-2
STLC (+) after thymidine synchronization
siControl
siCWC22#1
siCWC22#2
0 6 12 18 24
Time (h)
P/PM
mitotic slippage
mell death

B

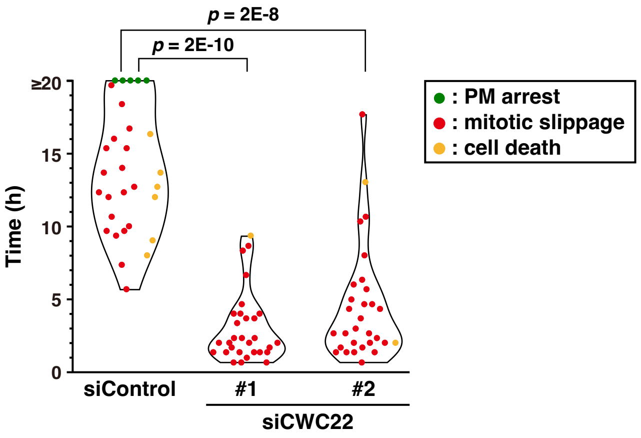
p = 2E-8
p = 2E-10
Time (h)
≥20
15
10
5
0
siControl
#1
#2
siCWC22
: PM arrest
: mitotic slippage
: cell death

Figure S2

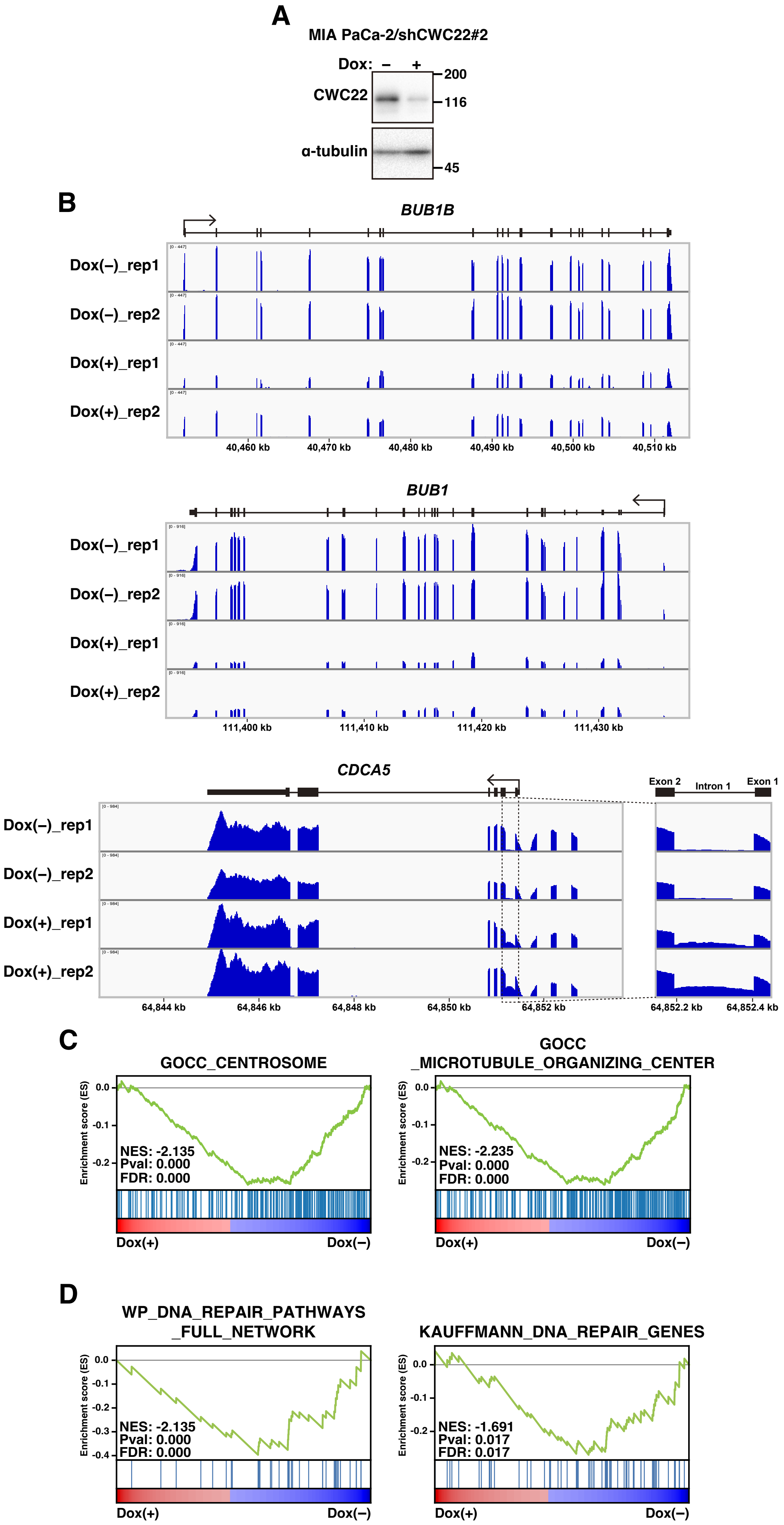

A
MIA PaCa-2/shCWC22#2
Dox: – +
CWC22
α-tubulin
200
116
45
B
BUB1B
Dox(–)_rep1
Dox(–)_rep2
Dox(+)_rep1
Dox(+)_rep2
40,460 kb
40,470 kb
40,480 kb
40,490 kb
40,500 kb
40,510 kb
BUB1
111,400 kb
111,410 kb
111,420 kb
111,430 kb
CDCA5
Exon 2
Intron 1
Exon 1
64,844 kb
64,846 kb
64,848 kb
64,850 kb
64,852 kb
64,852.2 kb
64,852.4 kb
C
GOCC_CENTROSOME
GOCC _MICROTUBULE_ORGANIZING_CENTER
Enrichment score (ES)
NES: -2.135
Pval: 0.000
FDR: 0.000
NES: -2.235
Pval: 0.000
FDR: 0.000
Dox(+)
Dox(–)
D
WP_DNA_REPAIR_PATHWAYS _FULL_NETWORK
KAUFFMANN_DNA_REPAIR_GENES
NES: -2.135
Pval: 0.000
FDR: 0.000
NES: -1.691
Pval: 0.017
FDR: 0.017

Figure S3

A

MIA PaCa-2/HA-Bub1

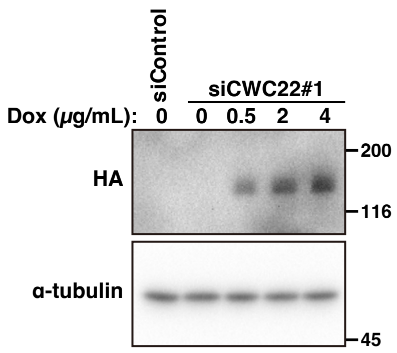

B

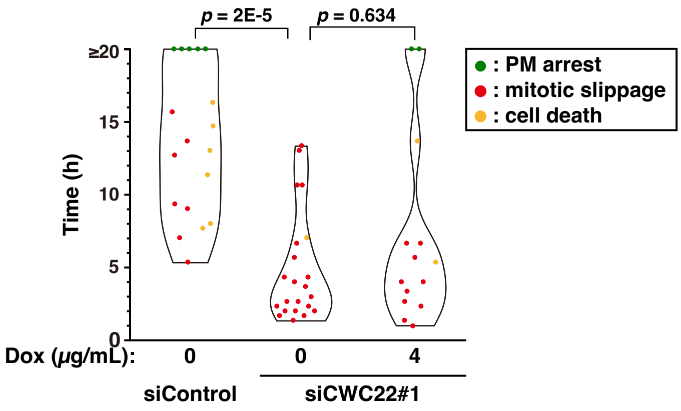

Figure S4

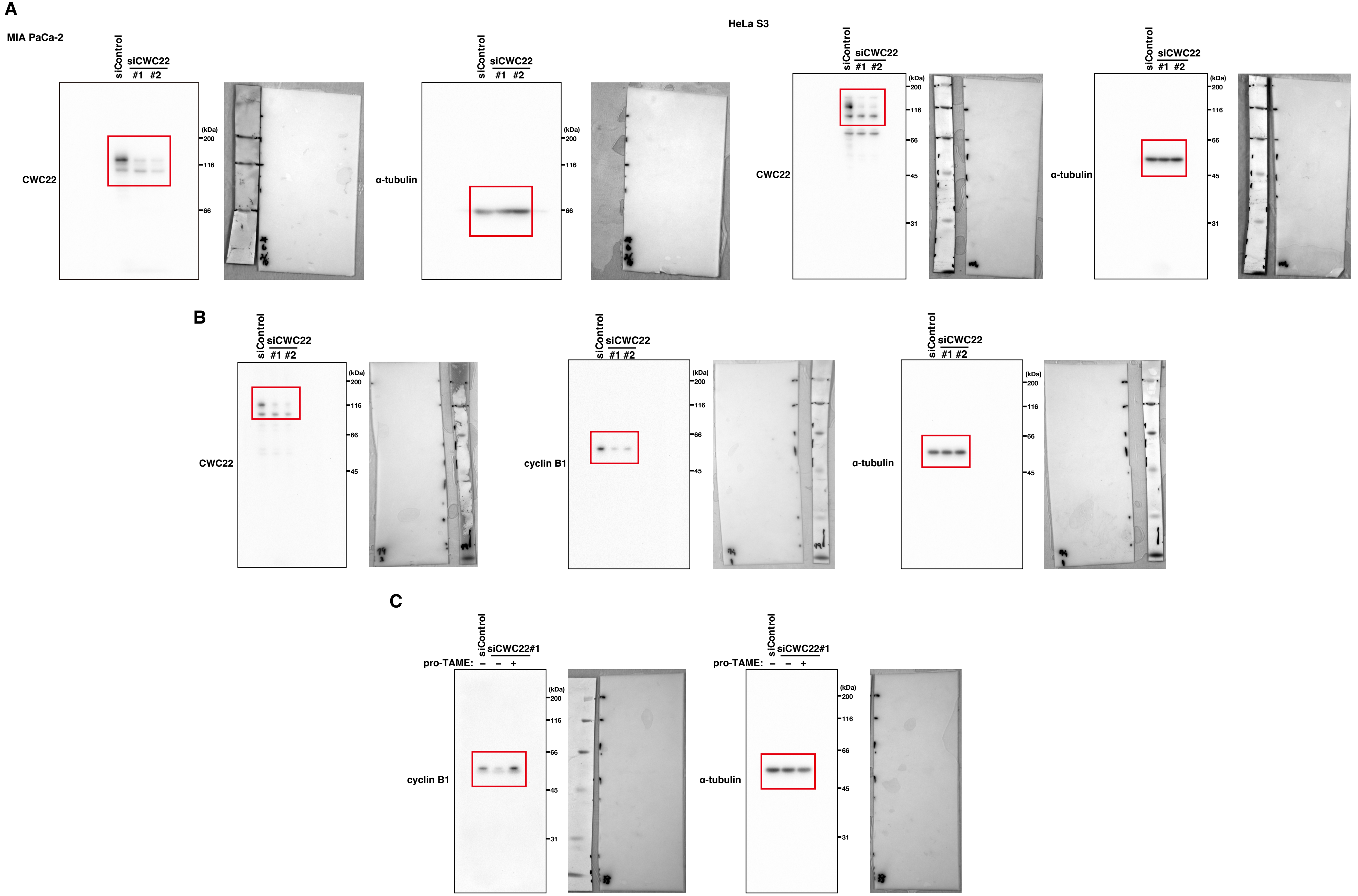
A
MIA PaCa-2
siControl
siCWC22
#1 #2
CWC22
(kDa)
200
116
66
α-tubulin
HeLa S3
45
31
B
cyclin B1
C
siCWC22#1
pro-TAME:
–
+

Figure S5-1

D

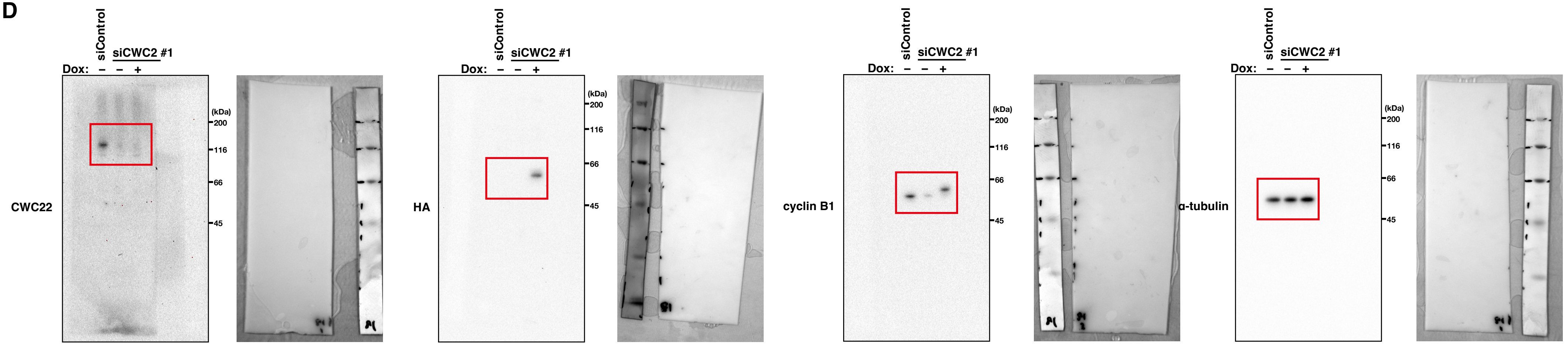

E

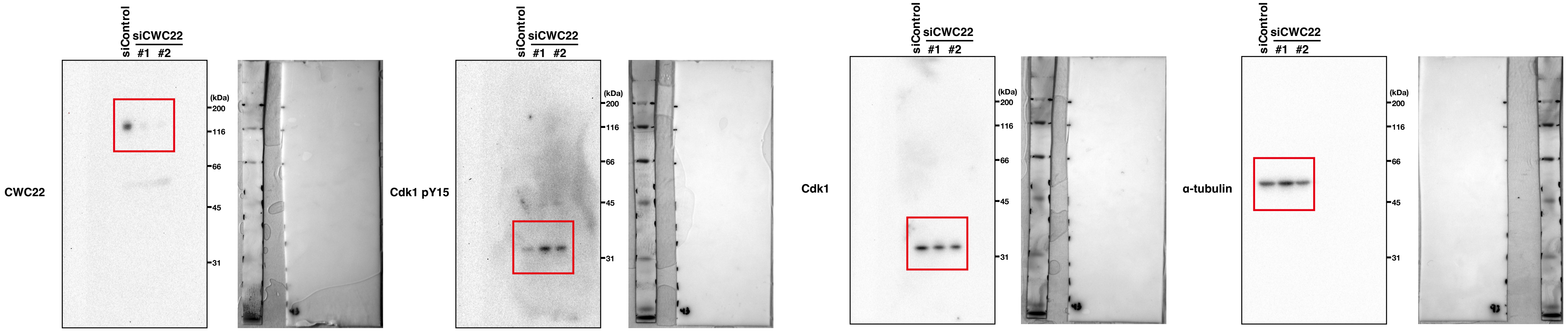

F

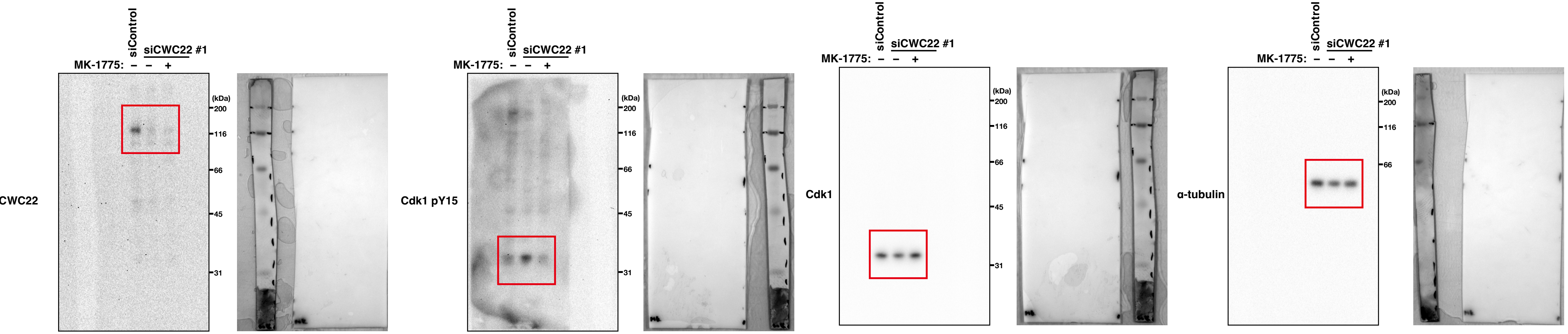

Figure S5-2

G

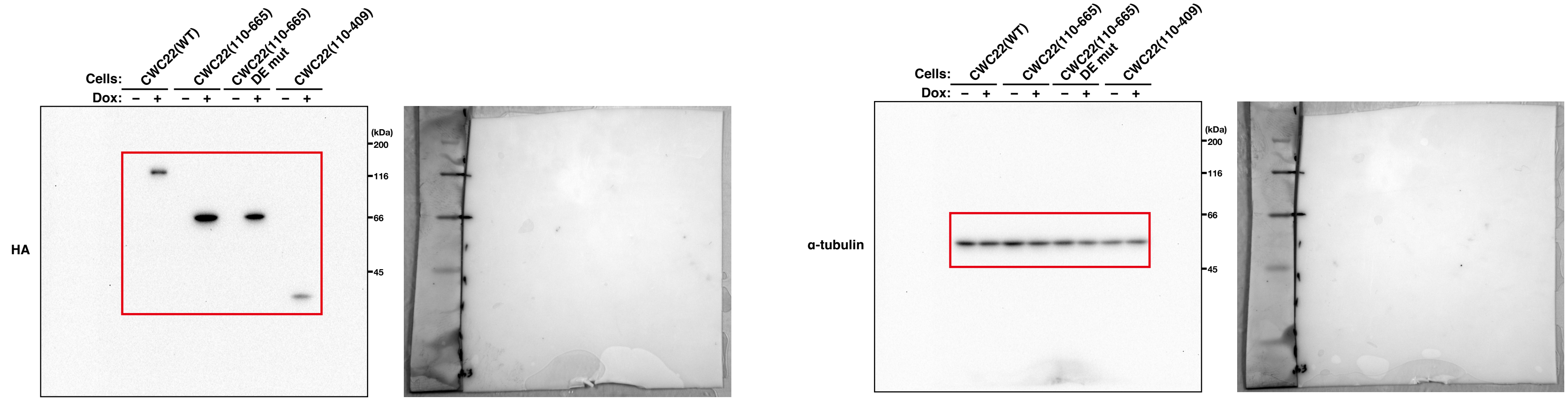

H

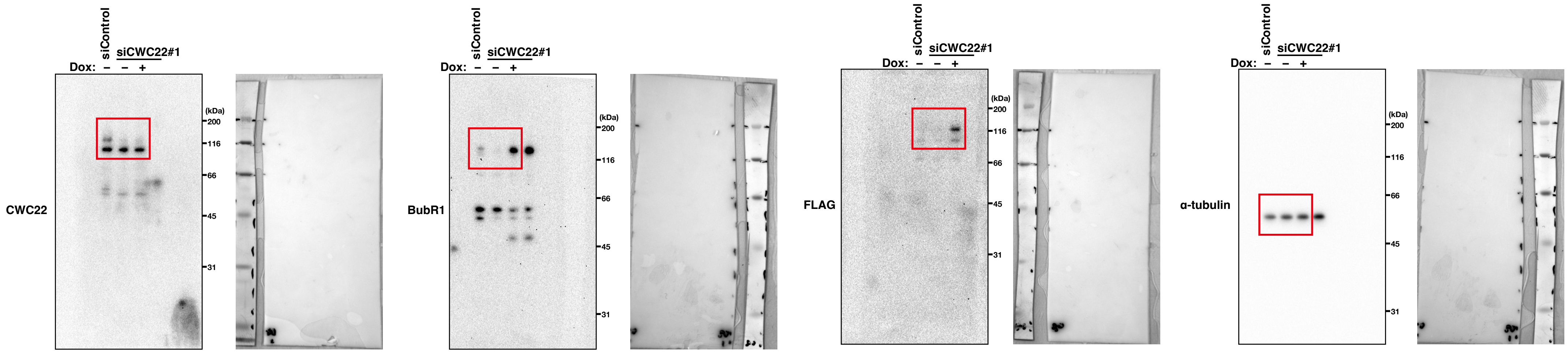

Figure S5-3

I

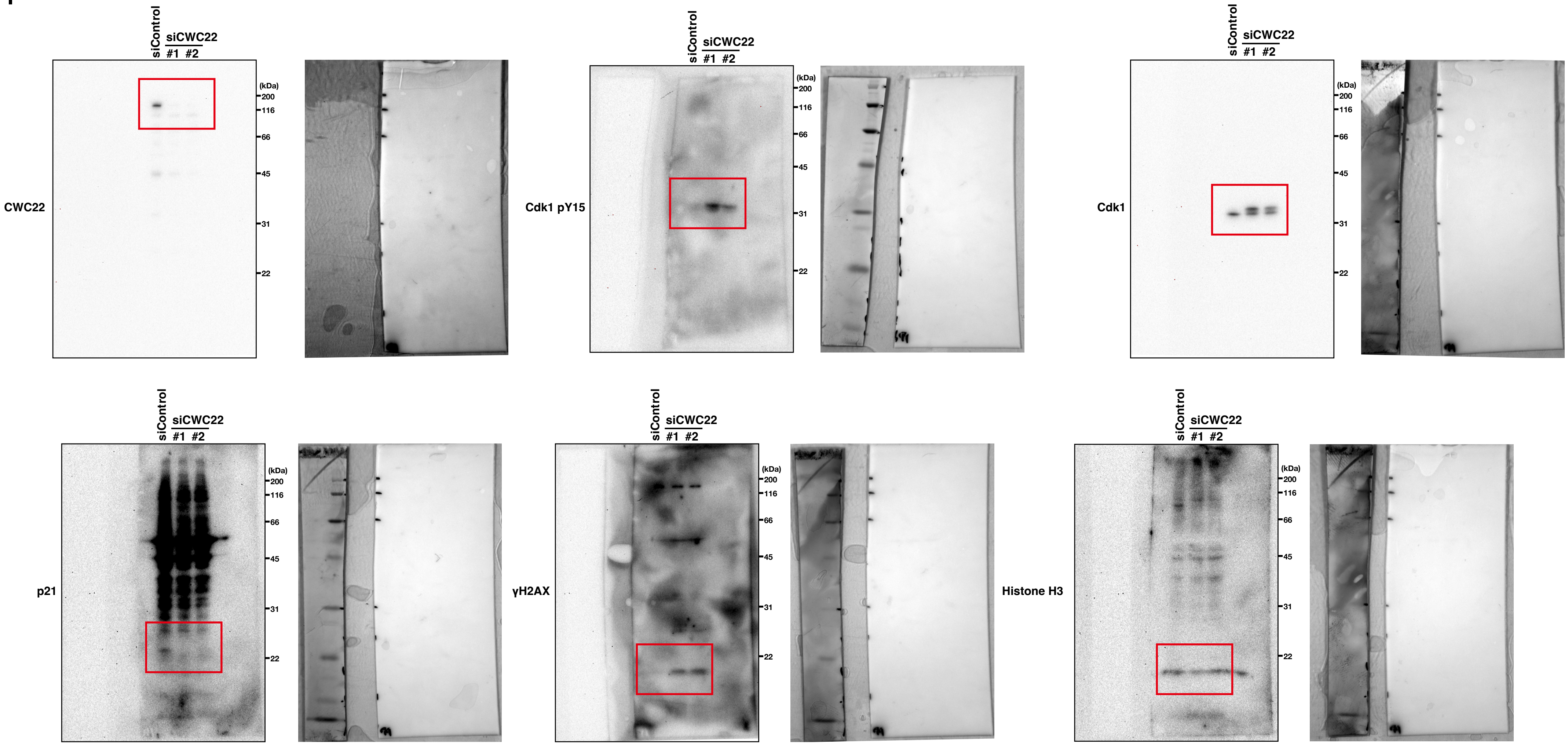

siControl
siCWC22
#1 #2
(kDa)
200
116
66
45
31
22
CWC22
Cdk1 pY15
Cdk1
p21
γH2AX
Histone H3

Figure S5-4

J

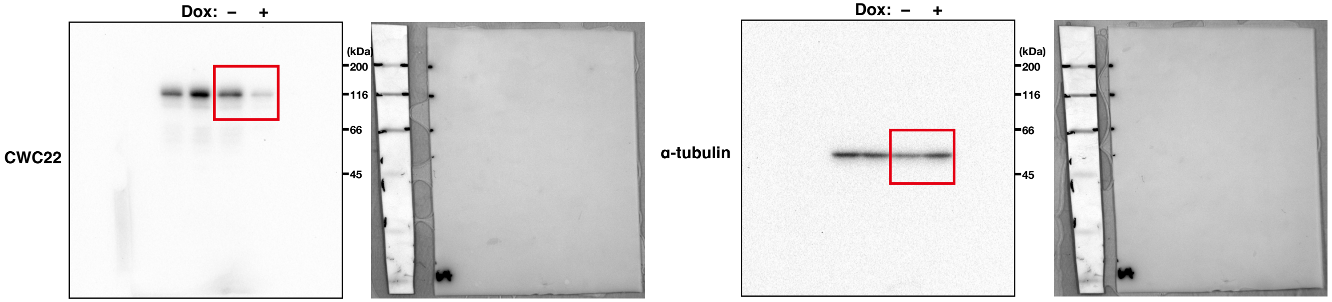

K

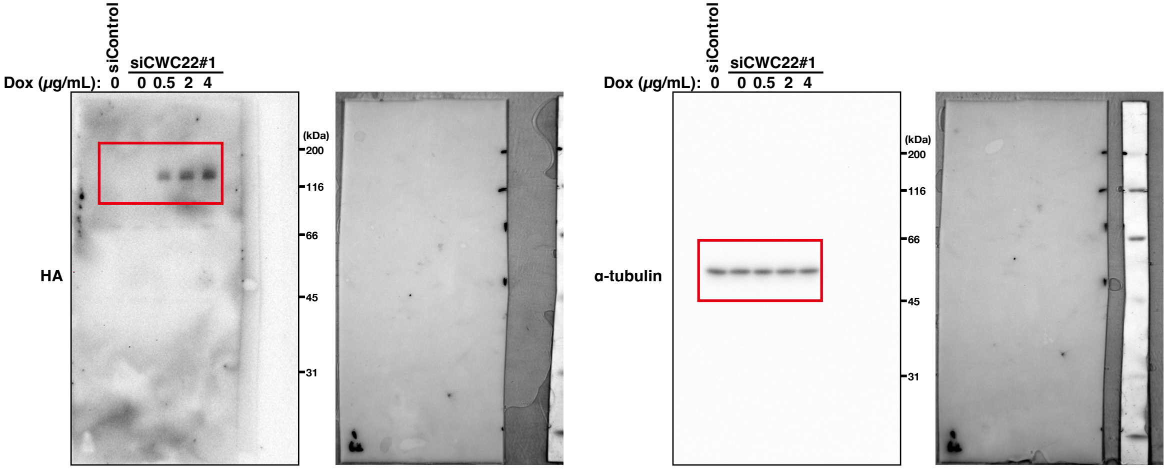

Figure S5-5
