# Supplementary material for: Targeting the splicing factor CWC22 induces mitotic slippage through repression of BubR1 expression and CDK1 activity in cancer cells
Source: J Biol Chem. 2026 Jan 12;302(3):111148. doi: 10.1016/j.jbc.2026.111148 (PMC12887411; doi:10.1016/j.jbc.2026.111148)
Supplement: Supplementary Material 2 [file mmc2.docx]

**Targeting the splicing factor CWC22 induces mitotic slippage through repression of BubR1 expression and CDK1 activity in cancer cells**

**Ryuzaburo Yuki^*^, Youhei Saito, and Yuji Nakayama^*^**

**Supporting information**

**Fig. S1. Cyclin B1 localization in G2 phase cells**

Related to Fig. 2C, representative images of prophase and G2 phase cells are shown. Scale bar, 10 µm.

**Fig. S2. CWC22 knockdown accelerates mitotic slippage under the synchronization condition**

MIA PaCa-2 cells or HeLa S3 cells were transfected with control siRNA (siControl) or CWC22-targeting siRNAs (siCWC22#1 and #2). At 19 h after siRNA transfection, the cells were treated with 4 mM thymidine for 20 h, washed with PBS(−), and cultured for a further 7 h. The cells were treated with 5 µM STLC for 1 h and monitored for 24 h by time-lapse imaging with 0.1 µM Hoechst 33342. (**A**) The duration of each mitotic phase is shown as indicated in Fig. 1D. (**B**) The duration of mitosis until mitotic slippage (red) or cell death (yellow) is shown as indicated in Fig. 2B (n = 30). p-values were determined using the Steel test.

**Fig. S3. RNA-seq analysis using MIA PaCa-2 cells expressing inducible CWC22#2 shRNA.**

(**A**) MIA PaCa-2/shCWC22#2 cells were treated with 4 µg/mL Dox for 48 h, and western blot analysis was performed with the indicated antibodies. (**B**) IGV genome browser tracks showing intron retention levels in *BUB1B*, *BUB1*, and *CDCA5* loci were shown in control and CWC22-knockdown mitotic cells. A magnified image of the intron 1 region of *CDCA5* was shown on the right. (**C**) GSEA plots for the gene set “GOCC_CENTROSOME” and “GOCC_MICROTUBULE_ORGANIZING_CENTER” between Dox-treated and untreated cells is shown. (**D**) GSEA plots for the gene set “WP_DNA_REPAIR_PATHWAYS _FULL_NETWORK” and “KAUFFMANN_DNA_REPAIR_GENES” between Dox-treated and untreated cells is shown.

**Fig. S4. Knockdown-rescue experiment of Bub1.**

(**A**) MIA PaCa-2/HA-Bub1 cells were transfected with control siRNA (siControl) or CWC22-targeting siRNAs (siCWC22#1) in the presence or absence of 0.5–4 µg/mL Dox. 36 h after transfection, the cells were treated with 5 µM STLC for 12 h. The mitotic cells were collected via mitotic shake-off, and western blot analysis was performed with the indicated antibodies. (**B**) At 39 h after transfection, the cells were treated with 5 µM STLC for 1 h and monitored for 24 h by time-lapse imaging with 0.1 µM Hoechst 33342. The duration of mitosis until mitotic slippage (red) or cell death (yellow) is shown as indicated in Fig. 2B. *p*-values were determined using Steel–Dwass test in panel (B).

**Fig. S5. Full-length blots.**
